# Medicinal-Cosmetic Potential of the Local Endemic Plants of Crete (Greece), Northern Morocco and Tunisia: Priorities for Conservation and Sustainable Exploitation of Neglected and Underutilized Phytogenetic Resources

**DOI:** 10.3390/biology10121344

**Published:** 2021-12-16

**Authors:** Soumaya Bourgou, Imtinen Ben Haj Jilani, Olfa Karous, Wided Megdiche-Ksouri, Zeineb Ghrabi-Gammar, Mohamed Libiad, Abdelmajid Khabbach, Mohamed El Haissoufi, Fatima Lamchouri, Vasileios Greveniotis, Manolis Avramakis, Stefanos Hatzilazarou, Ioannis Anestis, Georgios Tsoktouridis, Nikos Krigas

**Affiliations:** 1 Centre de Biotechnologie de Borj-Cédria, Laboratoire des Plantes Aromatiques et Médicinales, BP 901, Hammam-Lif 2050, Tunisia; ksouriwided@yahoo.fr; 2 Institut National Agronomique de Tunisie, Université de Carthage, 43 Avenue Charles Nicolle, Cité Mahrajène, Tunis 1082, Tunisia; imtinenbhj@yahoo.fr (I.B.H.J.); karous-olfa@hotmail.fr (O.K.); zghrabi@yahoo.fr (Z.G.-G.); 3 Laboratoire de Recherche Biogéographie, Climatologie Appliquée et Dynamiques Environnementales (BiCADE 18ES13), Faculté des Lettres des Arts et des Humanités de Manouba, Université de la Manouba, Manouba 2010, Tunisia; 4 Laboratory of Natural Substances, Pharmacology, Environment, Modelling, Health and Quality of Life (SNAMPOPEQ), Polydisciplinary Faculty of Taza, Sidi Mohamed Ben Abdellah University, B.P. 1223, Taza Gare, Taza 35000, Morocco; libiad001@gmail.com (M.L.); khamajid@hotmail.com (A.K.); mohamed.elhaissoufi1@usmba.ac.ma (M.E.H.); fatima.lamchouri@usmba.ac.ma (F.L.); 5 Laboratory of Ecology, Systematics and Biodiversity Conservation (LESCB), CNRST Labeled Research Unit Nº18, Department of Biology, Faculty of Sciences, Abdelmalek Essaâdi University, B.P. 2121, M’Hannech II, Tetouan 93000, Morocco; 6 Laboratory of Biotechnology, Conservation and Valorization of Natural Resources (BCVRN), Department of Biology, Faculty of Sciences Dhar El Mahraz, Sidi Mohamed Ben Abdellah University, B.P. 1796, Fès-Atlas 30003, Morocco; 7 Institute of Industrial and Forage Crops, Hellenic Agricultural Organization Demeter, 41335 Larisa, Greece; vgreveni@mail.com; 8 Natural History Museum of Crete, University of Crete, 71409 Heraklion, Greece; avram@nhmc.uoc.gr; 9 Laboratory of Floriculture, School of Agriculture, Aristotle University of Thessaloniki, 54124 Thessaloniki, Greece; hatzilaz@agro.auth.gr; 10 Institute of Plant Breeding and Genetic Resources, Hellenic Agricultural Organization Demeter, Thermi, P.O. Box 60458, 57001 Thessaloniki, Greece; ganestis3@gmail.com (I.A.); gtsok1@yahoo.co.uk (G.T.)

**Keywords:** biodiversity, ethnobotany, methodological scheme, phytochemistry, phytomedicine, traditional uses

## Abstract

**Simple Summary:**

Medicinal-aromatic plants are important sources of valuable products for human and animal health but also as ornamentals. Since very few studies exist on the domestication of neglected and underutilized plants (NUPs), we aimed to document and evaluate the medicinal-cosmetic potential of 399 local endemic NUPs confined to Crete (223), the Mediterranean coast-Rif of Morocco (94), and Tunisia (82). A new methodological scheme was developed through three multidisciplinary co-creative workshops by experts and was adjusted by end-users to point-scoring of nine attributes evaluating the potential of the targeted NUPs in the medicinal-cosmetic sector (results demonstrated as percentage of the maximum possible score). The latter were further linked and discussed with respect to feasibility and readiness timescale evaluations for sustainable exploitation of the focal NUPs. A great diversity of studied NUPs (30 taxa, 11 families) was detected as promising (scores 35–94.44%), and 8 taxa (half of which are threatened) were identified as the most prominent taxa for the medicinal-cosmetic sector. Although ex-situ conservation efforts and applied research work are needed to unlock the full potential of the local endemic NUPs, the sustainable exploitation of high-scored cases can be fastened through targeted species-specific research bridging extant research gaps and facilitating conservation and stakeholder attraction.

**Abstract:**

Medicinal-aromatic plants (MAPs) are important sources for the development of new valuable products of interest to human and animal health, and are also used as ornamentals for the horticulture industry. However, the increased global demand and the uncontrolled exploitation of these plants constitute a threat to their sustainability. To date, few scientific investigations have focused on MAPs valorization and their domestication. The purpose of this study was to evaluate for the first time the medicinal-cosmetic potential of 399 local endemic Mediterranean plants confined to Crete (223 taxa), the Mediterranean coast-Rif of Morocco (94), and Tunisia (82). The new methodological scheme was developed by experts through three multidisciplinary co-creative workshops and was adjusted by end-users to point-scoring of nine attributes evaluating the potential of the targeted neglected and underutilized plants (NUPs) in the medicinal-cosmetic sector. The results were demonstrated as percentage of the maximum possible score. These assessments were further linked and discussed with respect to feasibility and readiness timescale evaluations for sustainable exploitation of the focal NUPs. A great diversity of local endemic NUPs (30 taxa, 11 families) were associated with interesting medicinal-cosmetic properties (>35% up to 94.44%). Among them, 8 taxa showed the highest medicinal-cosmetic potential (>55% of maximum possible score), half of which are threatened with extinction. Although ex-situ conservation efforts and applied research work are needed to safeguard and unlock the full potential of the local endemic NUPs evaluated herein, the proposed multifaceted evaluation scheme revealed that some local endemic NUPs of the studied regions can be sustainably exploited in short- or medium-term, following successful examples of Cretan NUPs e.g., *Origanum dictramnus*. The sustainable exploitation of high scored taxa of the studied regions can be fastened through targeted species-specific research bridging extant research gaps and facilitating conservation and stakeholder attraction.

## 1. Introduction

Since ancient times, traditional herbal medicine has aimed to preserve health or to prevent and treat disease. Medicinal-Aromatic Plants (MAPs) constitute man’s first medicines and they still provide treatments to more than 80% of the world’s population [1]. In recent decades, herbal medicines have become a new trend with many people resorting to these products for treatment of various health challenges. Currently, more than 100 million Europeans use traditional or complementary medicine products [2], and global markets for medicines as well as health and wellness products are valued at $84 billion [3]. In animal husbandry, MAPs are used to date in alternative ways in attempts to avoid or reduce chemical drugs and antibiotics that raise concerns but also to support growth performance in farmed animals [4].

The beneficial remedial effects of MAPs are mainly due to the mixture of substances called secondary metabolites including isoprenoids (terpenoids), alkaloids, phenylpropanoids, and flavonoids (polyphenols). MAPs synthesize these metabolites through various physiological and biochemical processes in response to biotic and abiotic stresses [5]. Some of the most important anticancer and anti-infective drugs are plant-derived secondary metabolites such as emetine, taxol, vinblastine, and vincristine [6,7]. On the other hand, MAPs represent attractive ornamental plants for gardening or landscaping applications and are therefore well-appreciated by the ornamental-horticultural industry. These MAPs are also consumed on a daily basis by citizens in many parts of the world in culinary preparations and for beverages [8]. Therefore, there is an ongoing quest for new MAP crops with exciting characteristics and unique character, beneficial properties, high adaptability to diverse conditions, and low maintenance costs [8,9,10,11]. However, the sustainability of these natural resources is increasingly threatened by ill-advised exploitation, especially due to the globalization of MAPs’ trade [12,13,14]. In this respect, global research is more focused on the bioprospecting than the cultivation or domestication of plants with known medicinal potential [15]. To avoid the loss of these precious phytogenetic resources for human and animal life, health and well-being, the Rio convention on Biological Diversity signed in 1992 and the Nagoya protocol (its counterpart since 2010) defined the practices that should govern bioprospecting, also setting the basis of extant access and benefit sharing mechanisms (https://absch.cbd.int/, accessed on 13 December 2021).

The flora of Greece, Morocco, and Tunisia are quite rich and diversified, including approximately 6620, 5211, and 2700 taxa, respectively [16,17,18]. About 22% of the plant taxa of Greece are local endemics [16] with 223 of them confined only to Crete [19]. The flora of Tunisia hosts 82 endemic taxa, while that of Morocco hosts 879 local endemics [20] with 94 listed as local endemics of the Mediterranean coast-Rif of Morocco [21]. However, for the majority of these range-restricted local Mediterranean endemics, scarce information is available about their use in traditional medicine or as potential sources of raw materials of medicinal-cosmetic value. Unfortunately, only a small fraction of these unique plants is probably known by domestic people, and thus contributes to the uncharted domestic ethnobotanical knowledge of these regions, thus rendering these local endemics as neglected and underutilized plants (NUPs). Nevertheless, this unique natural heritage deserves to be further explored and documented, especially due to the scarcity of ethnobotanical studies dealing with local endemic NUPs. General ethnobotanical field studies have demonstrated to date that indigenous communities can be perceived in many regions as a noteworthy ancient resource of vanishing plant utilization and associated botanical-ecological knowledge, with this traditional knowledge on medicinal plants being mainly an asset of the elder people which is eroded among younger generations [1,2,4,12,22]. This trend needs to be improved and deserves to be explored in a more systematic way, while new research efforts should promptly document concrete uses of local endemic plants in different local communities to be able to contribute to the development of locally adapted and sustainable food as well as alternative and effective healthcare systems.

In this framework, the objective of this study is dual: Initially, to review for the first time old and recent published data related to the specific medicinal-cosmetic value of 399 local endemic plants of Crete (Greece), Rif-Mediterranean Coast of Morocco and Tunisia; and secondly, to assess their use as modern superfoods, functional foods or ethnobotanical resources with specific medicinal properties, known phytochemical composition, biological activities, and/or toxicity. The latter is envisaged in the framework of sustainability which allows potentially continuous yields of plant products without affecting neither the wild-growing populations of the species in concern nor their habitats. The procedure followed herein is aimed to prioritize local endemic NUPs per study region endowed with an interesting medicinal-cosmetic potential which could be valued adequately, exploited sustainably, and conserved effectively at the same time. The present study aims to serve as a baseline for further targeted phytochemical and pharmacological research, aiming to enhance the value of these local endemic resources, especially regarding the most threatened and/or overexploited MAPs.

## 2. Materials and Methods

### 2.1. Target Area and Focal Plants

The study area is the island of Crete (Greece), the Mediterranean coast-Rif of Morocco, and Tunisia (whole national territory) [19,20]. In total, 399 taxa (species and subspecies) are assessed in this study, and all are local endemic plants of specific Mediterranean regions, i.e., 223 single-island local endemic plant taxa of Crete, Greece [19], 94 single-region endemic taxa of the Mediterranean coast and Rif of Morocco; and 82 single-country endemic taxa of Tunisia [20].

### 2.2. Evaluation Levels

This study integrates part of the new approach for the multifaceted evaluation of neglected and underutilized plants (NUPs) in different economic sectors as developed in the frame of the MULTI-VAL-END project (ARIMNet2) (for full description, see Krigas et al. [8], for specialization in the agro-alimentary sector, see Libiad et al. [22]). As an outcome of multidisciplinary and co-creative research (13 scientists from Greece, Morocco, and Tunisia, all authors of this study), point-scoring of nine selected attributes were used to document in this study the specific potential of the local endemic NUPs in the medicinal-cosmetic sector (Level I evaluation). The multidisciplinary consortium decided collectively the individual attributes for the medicinal-cosmetic sector to be used for the evaluation of taxa; defined the typology of attributes used for evaluation (sector-specific or inter-sectorial); selected the data sources to be consulted for documentation (one to four types per selected attribute); defined the scaling for scoring in each Level I attribute (how many possible scores and value definitions), and the scoring directionality per attribute (lower to higher values) on the basis of quality and quantity of extant information retrieved for the focal NUPs (see Supplementary Material S1). The medicinal-cosmetic potential of the studied 399 local endemic NUPs was assessed using a point scoring system with nine sector-specific attributes as outlined in Table 1. In total, four attributes allowed a four-grade scale (4 possible scores); two attributes were assigned with a five-grade scale to generate score values, and three attributes involved seven possible scores (Table 1). Characteristic examples of scoring for the studied taxa along with guidelines and sources consulted to generate the taxon-specific scores are given explicitly in Supplementary Material S1. Upon scoring completion per taxon, the sum of scorings for all attributes was calculated and it was expressed as relative percentage (%) of the maximum possible score that could be generated in the medicinal-cosmetic sector, i.e., sum of maximum scores for all attributes. To illustrate the most interesting/promising NUPs per country for the medicinal-cosmetic sector, three lists of hierarchically ranked taxa per country were produced (see Supplementary Material S2). The previously published methodological scheme [8,22] is specialized herein for the first time regarding the medicinal-cosmetic sector with new assessments. These taxon-specific assessments were linked with previous assessments such as feasibility (Level II evaluation) and readiness timescale for sustainable exploitation (Level III evaluation). The latter (Level II and III evaluations) are envisaged herein for the first time in a sector-specific mode, i.e., specifically for the medicinal-cosmetic sector.

The feasibility of each taxon for the sustainable exploitation in the medicinal-cosmetic sector (Level II evaluation) was assessed on the basis of 12 attributes described in previous own studies [8,22]. These selected attributes were employed as prerequisites of common interest [8,22] since they facilitate the sustainable exploitation of the target taxa (Level II evaluation) in the medicinal-cosmetic sector as well. The readiness timescale for value chain creation regarding the focal NUPs (Level III evaluation) was based on the SWOT (Strengths, Weaknesses, Opportunities, Threats) analysis and the gap analysis described in own previous research [8,22] which is specialized herein for the medicinal-cosmetic sector.

### 2.3. Statistical Analysis

To explore the way in which the different medicinal attributes and concomitant scorings of the focal NUPs are grouped per study region, we performed complete linkage hierarchical cluster analyses with 1-Pearson r distance measure, based on the scores generated for the studied local endemic taxa. More specifically, this was possible to be achieved for 9 (all), 8 (all but one), and 7 (all but two) of the medicinal-cosmetic attributes related to the local endemic plants of Crete, Mediterranean coast-Rif of Morocco, and Tunisia, respectively, due to the fact that some of the attributes had to be excluded since no available data were found or the same scoring was generated for all taxa.

## 3. Results

### 3.1. Clusterings of Medicinal-Cosmetic Attributes per Region

**Figure 1:**
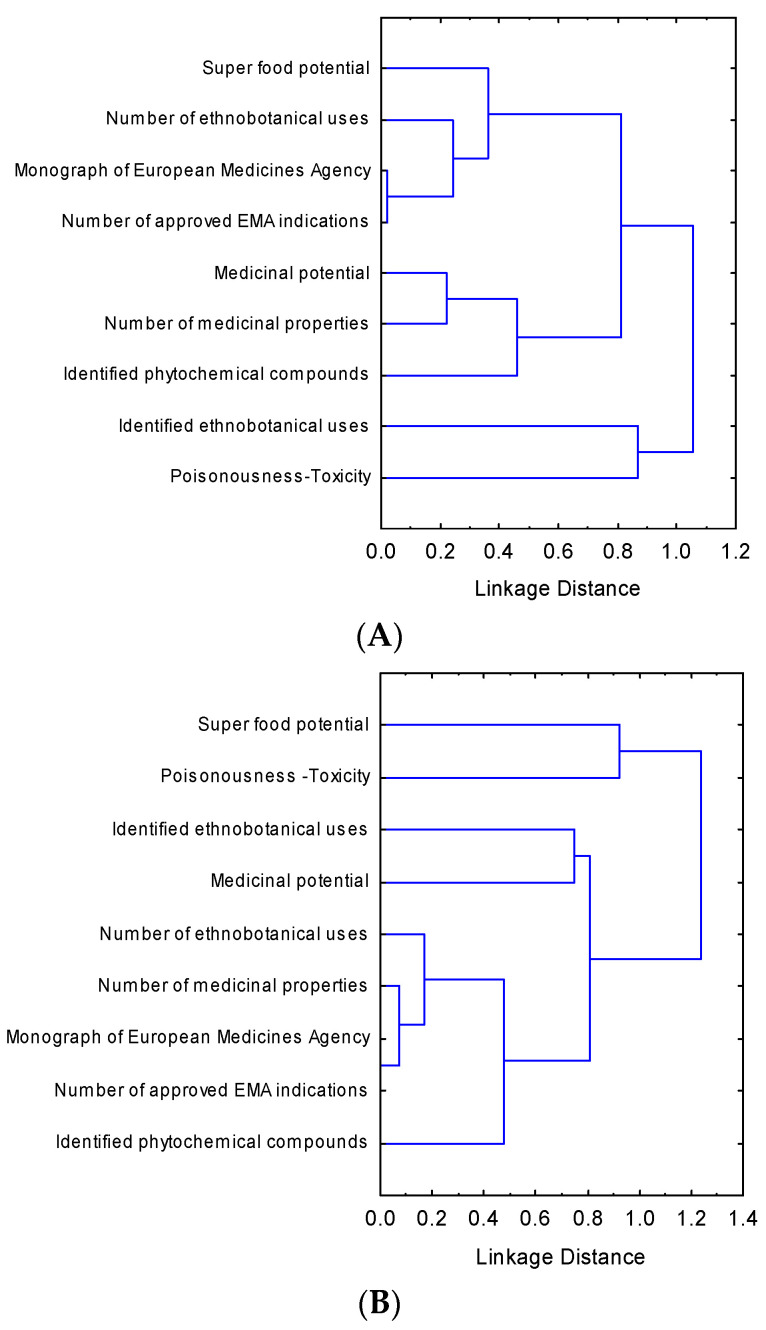

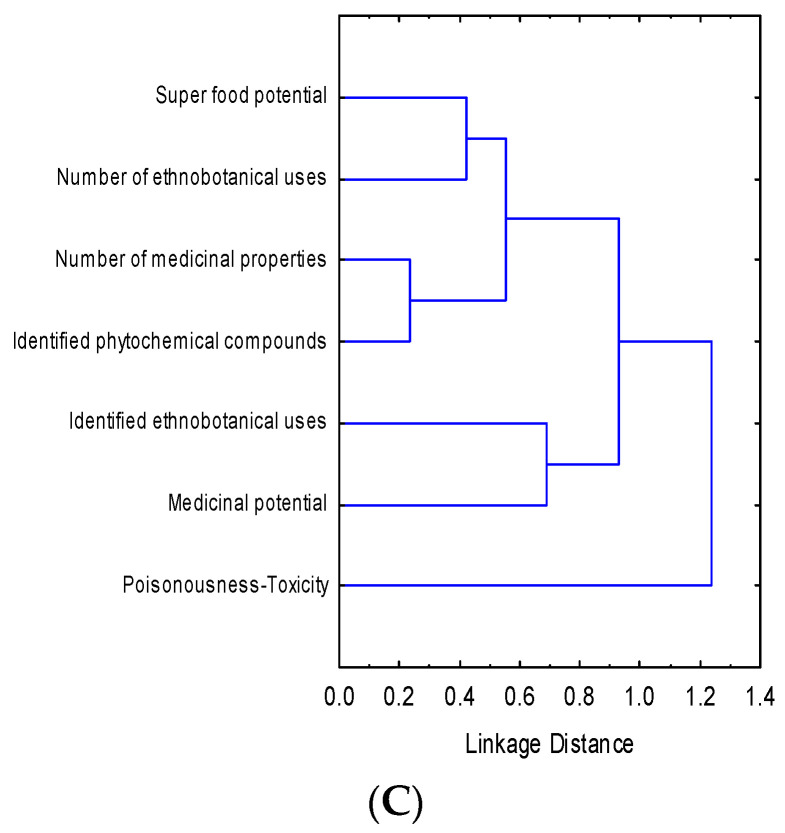
Graph of hierarchical clustering of medicinal-cosmetic attributes (complete linkage, 1-Pearson r distance) based on the score values of the 223 local endemic plants of Crete (**A**), 94 local endemic taxa of Mediterranean coast-Rif of Morocco (**B**), and 82 local endemic taxa of Tunisia (**C**).

The results of the hierarchical cluster analyses of the medicinal-cosmetic attributes and their scores (Figure 1) showed that for the case of the studied Cretan NUPs, the attributes “super food potential”, “number of ethnobotanical uses”, “monograph or European Medicines Agency (EMA)” (proxies of officially approved uses as traditional herbal medicine) were clustered together with “number of approved EMA indications” (proxy of associated health-claiming properties). Another cluster was formed by the attributes “medicinal potential”, “number of medicinal properties”, and “identified phytochemical compounds” (proxies of medicinal-cosmetic documentation), while a third cluster was formed by the attributes “identified ethnobotanical uses” (proxy of ethnobotanical values) and “poisonousness/toxicity” (proxy of safety).

In the case of the NUPs of Mediterranean coast-Rif of Morocco, the attributes “poisonousness/toxicity” and “super food potential” were grouped together and were separated from other ones indicating distinct scorings. The attributes “identified ethnobotanical uses” and “medicinal potential” formed another distinct subcluster and were grouped together with the subcluster of the attributes related to medicinal-cosmetic documentation and beneficial health effects such as “number of ethnobotanical uses”, “number of medicinal properties”, “monograph of EMA”, number of approved EMA indications”, and “identified phytochemical compounds”.

In the case of the studied Tunisian NUPs, the attributes “super food potential” and “number of ethnobotanical uses” formed a distinct subcluster and were clustered together with the subcluster “number of medicinal properties” and “identified phytochemical compounds”. Another subcluster was formed by the attributes “identified ethnobotanical uses” and “medicinal potential”. The attributes “poisonousness/toxicity” was discerned from all other attributes indicating again a distinct scoring.

### 3.2. Overall Diversity of Local Endemic NUPs with Interesting Medicinal Potential

The evaluation of the potential of the studied local endemic NUPs in the medicinal- cosmetic sector for the three studied regions together has outlined 30 local endemic taxa of 11 families which exhibit interesting medicinal potential (>35% of maximum possible score), i.e., 18 Cretan taxa in seven families, five taxa in the case of the Mediterranean coast and Rif of Morocco (all in different families), and seven Tunisian taxa of three families. Generally, Lamiaceae was the most prominent plant family for the studied regions, including almost half of the plants of interest (in total, 13 taxa: eight Cretan, four Tunisian and one from Mediterranean coast-Rif of Morocco) and mainly comprised members of the genera *Teucrium* (5 taxa) and *Origanum* (3). The second most represented family among local endemic NUPs of interest was Asteraceae with five taxa (three Cretan and two Tunisian NUPs). The remaining seven families were represented in only one of the studied regions and included a member of a single genus.

### 3.3. NUPs of Different Regions

Supplementary Material S1 provides examples of individual scoring of target taxa per medicinal-cosmetic attribute from all studied regions with explanations of how scoring was performed based on various sources after consultation. The overall evaluation of the medicinal-cosmetic potential (Level I) of the local endemic taxa per studied region is presented in Supplementary Material S2 as percentages of the maximum possible score achieved, i.e., sum of all taxon scorings for all attributes. These assessments are further linked with taxon score values regarding feasibility for sustainable exploitation (Level II evaluation) and characterization about readiness timescale for value chain creation (Level III) from previous own investigation [8].

#### 3.3.1. Cretan NUPs

Among the 223 taxa of Crete, 18 were associated with promising potential (>35%) in the medicinal-cosmetic sector, including eight Lamiaceae members of the genera *Origanum* (2), *Teucrium* (2), *Thymbra* (1), *Sideritis* (1), *Phlomis* (1), and *Calamintha* (1), and three Asteraceae members belonging to the genera *Helichrysum (*2) and *Onopordon* (1). The remaining were members of Campanulaceae and Hypericaceae (two in each case) or of Apiaceae, Asparagaceae, and Boraginaceae (one per case). For the remaining evaluated taxa (*n* = 205), the scores were comparatively very low (<35%). Zero scoring was assigned to two poisonous Cretan taxa, e.g., *Vincetoxicum creticum* Browicz and *Securigera globosa* (Lam.) Lassen (see Supplementary Material S2).

**Figure 2:**
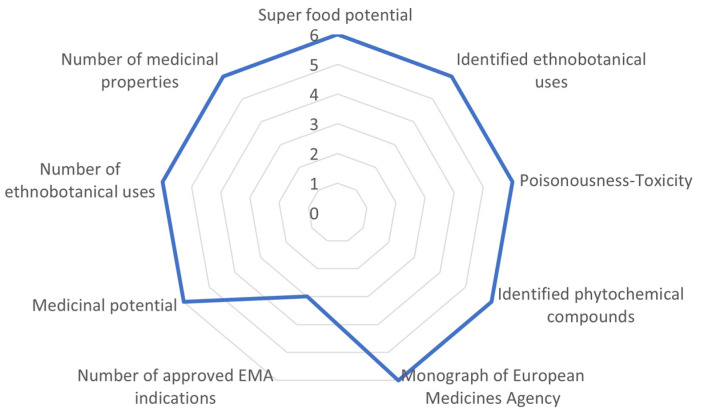
Evaluation example of *Origanum dictamnus* (Cretan endemic) scored for nine medicinal-cosmetic attributes reaching 94.44% of the optimum possible score. This example is hierarchically ranked in the highest class (>70%) among 399 studied taxa. For attributes and scoring, see Table 1.

Among the evaluated Cretan NUPs, two taxa of the Lamiaceae family showed a very high medicinal-cosmetic potential with scores greater than 70%, i.e., *Origanum dictamnus* L. (94.4%) and *Sideritis syriaca* L. subsp*. syriaca* (81.5%). The partial scorings of *O. dictamnus* per attribute is illustrated in Figure 2. In addition, *Origanum microphyllum* (Benth.) Vogel also showed an interesting medicinal-cosmetic potential with score of 59.3%. In total, another 15 taxa were classified with average to low score (35–50%) including *Phlomis lanata* Willd. (46.30%) and *Thymbra calostachya* (Rech.f.) Rech. f. (48.15*%), Calamintha cretica* (L.) Lam. (46.30%), *Teucrium alpestre* Sm. (40.74%), and *T. cuneifolium* Webb (40.74%) (all Lamiaceae).

#### 3.3.2. NUPs of the Mediterranean Coast-Rif of Morocco

Among the 94 NUPs of Mediterranean coast-Rif of Morocco, five taxa were outlined as promising (>35%) in the medicinal-cosmetic sector belonging to the genera (Family) *Abies* (Pinaceae), *Centaurium* (Gentianaceae), *Malva* (Malvaceae), *Origanum* (Lamiaceae), and *Rumex* (Polygonaceae). For the remaining taxa (*n* = 89), the scores were rather very low (<27.8%) and the lowest ones were assigned to three *Festuca* members (Poaceae), i.e., *F. embergeri* (Litard. & Maire) Romo*, F*. *fontqueriana* (St.-Yves) Romo, and *F. rifana* Litard. & Maire (each 3.7%).

**Figure 3:**
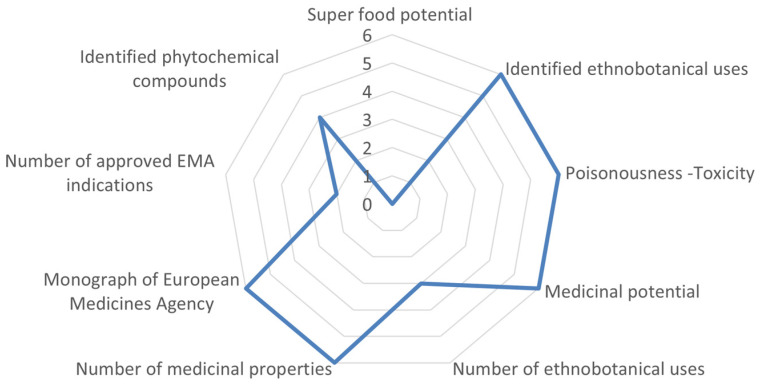
Evaluation example of *Centaurium eythraea* subsp. *bifrons* (north Moroccan endemic) scored for nine medicinal-cosmetic attributes reaching 72.2% of the optimum possible score. This example is hierarchically ranked in the above-average to high class. For attributes and scoring, see Table 1.

The highest medicinal-cosmetic potential in the case of northern Morocco was detected for *Centaurium erythraea* Rafn subsp. *bifrons* (Pau) Greuter which ranked above average with a score of 72.22% (Figure 3), followed by *Origanum elongatum* (Bonnet) Emb. & Maire that scored above average to high (55–70%). Three taxa scored below average to low (35–50%), i.e., *Abies marocana* Trab. of Pinaceae (44.44%), *Malva vidalii* (Pau) Molero & J. M. Monts. of Malvaceae, and *Rumex brachypodus* Rech. f. of Polygonaceae (35.2%).

#### 3.3.3. Tunisian NUPs

Among the 82 NUPs of Tunisia, seven taxa were detected with interesting potential (>35%) in the medicinal-cosmetic sector, four of which belong to Lamiaceae (three *Teucrium* spp. and one in genus *Marrubium*). The other interesting Tunisian NUPs belong to Asteraceae family (two taxa of the genera *Calendula* and *Artemisia)*, while Apiaceae was represented by a single taxon of genus *Ferula*. For the remaining taxa (*n* = 75), the score was comparatively very low with values < 35% (Supplementary Material S2).

**Figure 4:**
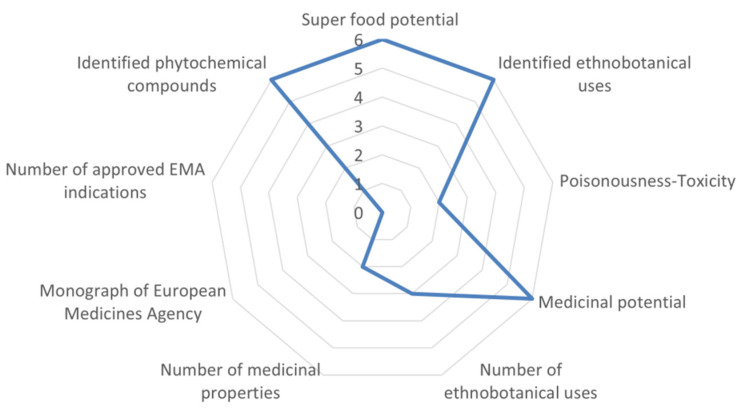
Evaluation example of *Teucrium alopecurus* (Tunisian endemic) scored for nine medicinal-cosmetic attributes, reaching 57.4% of the optimum possible score. This example is hierarchically ranked in the above-average to high class. For attributes and scoring, see Table 1.

None of the Tunisian taxa was ranked in the highest class in terms of strong potential (>70%) in the medicinal-cosmetic sector. *Teucrium alopecurus* Noë showed comparatively the most interesting medicinal-cosmetic potential among Tunisian NUPs (57.41%) and its scoring is illustrated in Figure 4. *Teucrium sauvagei* Le Houér (53.70%) and *Artemisia campestris* L. subsp. *cinerea* Le Houér (50%) of Asteraceae ranked rather average with regards to the optimum possible score, while another four taxa were scored with average to low scores (35–50%).

### 3.4. Feasibility and Readiness for Sustainable Exploitation of the Top NUPs in the Medicinal-Cosmetic Sector

This study showed that *Origanum dictamnus* (wild-growing Cretan endemic and locally cultivated MAP crop in Crete) represents the most promising case of endemic NUP among the 399 taxa evaluated in this study. This is due to the very high potential (>70%) in the medicinal-cosmetic sector coupled with very high feasibility for sustainable exploitation (91.67%, Supplementary Material S2). These scores actually outline the extant value chain and the sustainable commercial exploitation associated with this taxon, as already achieved mostly in Crete but also abroad [8]. Moreover, the Cretan *Sideritis syriaca* subsp. *syriaca* is also ranked very high both in terms of medicinal-cosmetic potential (81.48%) and in sustainable exploitation feasibility score (66.67%) (Supplementary Material S2). This taxon has already become a pilot crop locally in Crete with established value chain based on ancient traditions and modern market channels [8] as well as on targeted research recently accomplished [23].

*Origanum microphyllum* (Cretan NUP) followed by *Artemisia campestris* subsp. *cinerea* (Tunisian NUP) ranked in the above average class (50–55%) in terms of medicinal-cosmetic potential (59.26% and 50.00%, respectively). Consequently, the value chains for these taxa can be considered as reachable in the medium-term which means that they probably have the chance to become new MAP crops in the near future if facilitated by targeted research to stakeholder attraction.

However, among the top-evaluated Moroccan (Mediterranean coast and Rif) endemics in terms of medicinal-cosmetic potential, no taxon is evaluated in the highest class (>70%) or in above-average to high positions (>55–70%) in terms of sustainable exploitation feasibility. For example, although *Centaurium erythraea* subsp. *bifrons* (72.22%) scored very high and *Origanum elongatum* (55.55%) scored in above average to high class in terms of potential in the medicinal-cosmetic sector, these two taxa have ranked only below-average in terms of sustainable exploitation feasibility (40.28% for the first and 36.11% for the second). In the same trend, other interesting *Teucrium* taxa such as *T. alopecurus* and *T. sauvagei* from Tunisia that scored in terms of potential in above average positions (57.41% and 53.70%, respectively), they have actually scored very low (<35%) in terms of sustainable exploitation feasibility (27.78% and 22.22%, respectively). These results indicate substantial gaps which must be bridged for the exploitation of these taxa with interesting medicinal-cosmetic potential, and therefore subsequent efforts should be undertaken to facilitate their sustainable exploitation.

## 4. Discussion

Despite advances in documented ethnopharmacological knowledge related to MAPs, research focus regarding local endemic MAPs is still underway and limited, and therefore, their potential is rather ill-explored to date [24]. Despite the fact that local endemic NUPs may have possible beneficial effects for human and animal health, there is significant lack of scientific information available for such taxa or this knowledge is widely scattered in various literature sources and in different languages, thus making them truly ignored and non-utilized plants [25]. Coordinated research efforts in this direction may help to better explore and document the potential of local endemic MAPs that are considered as NUPs unlocking possibilities to contribute to the development of locally adapted and sustainable food resources as well as to detect alternative and effective traditional healthcare systems.

The current study introduced a new methodology for the multifaceted medicinal-cosmetic evaluation of NUPs, focusing on 399 unique floristic elements (single-region or single-country endemic plants) of three Mediterranean regions (Crete, Greece; Mediterranean coast-Rif of Morocco; Tunisia). This investigation highlights for the first time the potential of these rare endemic and unexplored NUPs in the medicinal-cosmetic sector, and explores the possibilities for their sustainable exploitation. Through hierarchical ranking procedures, our study outlined 30 top-evaluated cases, thus allowing the identification of the most interesting or promising local endemic NUPs per studied region. Overall, 30 NUPs have emerged as promising in the medicinal-cosmetic sector, originating from and confined to Crete (18 taxa), Tunisia (7), and the Mediterranean coast and Rif of Morocco (5). These prioritized top NUPs mainly belong to Lamiaceae (*Origanum dictamnus*, *Sideritis syriaca* subsp. *syriaca* and *Origanum microphyllum* for Crete, and *Teucrium alopecurus* and *T. sauvagei* for Tunisia); Gentianaceae (*Centaurium erythraea* subsp. *bifrons* from Morocco), and Asteraceae (*Artemisia campestris* subsp. *cinerea* for Tunisia) (Supplementary Material S2).

The promising local endemic NUPs include cases of NUPs with recognized ethnobotanical uses such as members of genera *Origanum*, *Sideritis, Campanula,* and *Centaurea*, as well as MAPs with reported value and properties, e.g., the local endemic NUPs of the genera *Origanum*, *Sideritis,* and *Teucrium* (Supplementary Material S1 and references therein). For example, the medicinal uses of two of these taxa are supported by finalized monographs and approved medicinal indications by the European Medicines Agency (*S. syriaca* subsp. *syriaca* and *O. dictamnus*). Furthermore, several taxa of the local endemic NUPs are geographically isolated subspecies (*Centaurium erythraea* subsp. *bifrons*) or close relatives (members of the same genus) of important MAPs with finalized monographs and approved medicinal indications by the European Medicines Agency [13], such as *Centaurium erythraea*, *Artemisia absinthium* L., *Hypericum perforatum* L., *Marrubium vulgare* L., *Origanum majorana* L., *Polygonum aviculare* L., *Salvia officinalis* L., *Salix* spp., *Verbasum* spp., and *Viola* spp.

The medicinal-cosmetic potential also reflects the richness of bioactive compounds, which is known to be prominent in members of *Origanum*, *Artemisia,* and *Teucrium* (Supplementary Material S1 and references therein). Several members of the genera *Origanum* and *Sideritis* are important in medicine because they are associated with local traditional uses and biological activities (antimicrobial, antifungal, antioxidant, antibacterial, antiparasitic, antihyperglycemic, etc.) which are scientifically validated [26,27]. In the three studied regions, three *Origanum* taxa and five taxa of the genus *Sideritis* are local endemic NUPs [8], i.e., *O. dictamnus* and *O. microphyllum* from Crete and *O. elongatum* from Morocco as well as *S. syriaca* subsp. *syriaca* from Crete, *S. romoi* Peris & al. and *S. arborescens* Salzm. ex Benth. subsp. *maireana* (Font Quer & Pau) Socorro & Arrebola from Morocco, and *S. tunetana* (Murb.) Obón & D. Rivera from Tunisia. Despite this fact, only the three Cretan endemics (*S. syriaca* subsp. *syriaca*, *O. dictamnus* and *O. microphyllum*) are currently assessed as most promising due to established value chains [8,22] (Supplementary Material S2). However, with targeted research and stakeholder attention *O. elongatum, S. romoi, S. arborescens* subsp. *maireana,* and *S. tunetana* may prove to have similar potential with the Cretan taxa of the same genus that are associated already with extant value chains.

### 4.1. Top-Evaluated NUPs of Crete

**Figure 5:**
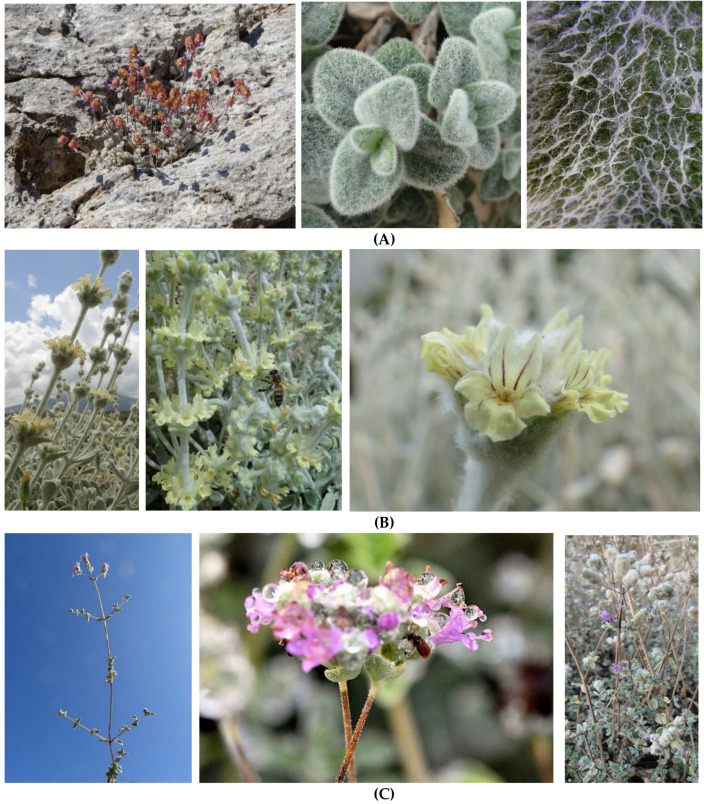
Top-evaluated local endemic plants of Crete (Greece) threatened with extinction with strong medicinal potential: (**A**) *Origanum dictamnus* in its wild habitat on Mt. Psiloritis (left) with strongly pubescent leaves (middle) and arachnoid indumentum (left) (Photo: T. Schizas, reproduced with permission); (**B**) Flowering shoots of *Sideritis syriaca* subsp. *syriaca* (left) with distant inflorescence verticillasters (middle) of bilabiate flowers (corollas with brown stripes on upper lip, right); (**C**) *Origanum microphyllum* shoots with small leaves at the beginning of flowering (left), partial inflorescence verticillasters (Photo: A. Papastergiopoulos, reproduced with permission) and appearance with comparatively larger leaves at the end of flowering.

Dittany of Crete (*Origanum dictamnus*, Figure 5A) is a perennial chasmophyte of several gorges and rock crevices of the island of Crete [28] which is assessed as Near Threatened [29] or Endangered [30] against the IUCN criteria, and locally cultivated [27]. It is protected by the Greek Presidential Decree 67/1981 while its traditional use as herbal medicine is supported by a European Medicines agency monograph [27,31]. The essential oil of *O. dictamnus* may heal wounds, can soothe pain, and seem to facilitate childbirth, presenting antirheumatic, oxytocic, stomach, and vulnerary activities [26]. Recent studies have also revealed remarkable antimicrobial and antioxidant activities of the essential oil of *O. dictamnus* as well as potency of cytotoxicity against HepG2 cells [32]. The phenol carvacrol which is often the main constituent of the plant’s essential oil represents an effective and inexpensive source of powerful natural antimicrobial agent with beneficial properties for human and animal health which can be incorporated into food systems or culinary preparations [4,27,31]. Eventually, the essential oils of *O. dictamnus* may also find similar applications in the future as those of famous Greek oregano [*Origanum vulgare* L. subsp. *hirtum* (Link) A. Terracc.], thus enhancing the potential of *O. dicatmnus* to wider usability further supporting subsistence of local cultivations in Crete.

Cretan mountain tea (*Sideritis syriaca* subsp*. syriaca*) is one of the 150 species in the genus *Sideritis* L. [33]. The perennial taxa of this genus have aroused great scientific interest to date resulting in several studies and a European Union Herbal Monograph [26]. They have been used as traditional medicinal plants for thousands of years and to date dosages and methods of preparation are well-defined and sufficiently documented [26]. Perennial *Sideritis* spp. present a wide range of biological activities such as antioxidant, anti-inflammatory, antivirus, anticancer, hepatoprotective, antispasmodic, analgesic, neuroprotective, as well as efficacy against diseases related to the central nervous system and urinary system [26,33,34,35,36]. These recognized activities are based on pharmacological characteristics which are mainly due to the presence of terpenes, flavonoids, phenylethanoid glucosides, phenolic acids, and essential oil [26,34]. The infusion of *S. syriaca* subsp. *syriaca* (traded as Mountain Tea, Cretan Mountain Tea, Greek Mountain Tea, Ironwort, or Malotira) (Figure 5B) has been listed by the European Medical Agency [26] as a traditional medicine for relief of mild gastrointestinal discomfort and against colds. The hexane compound, extracted from the powder of this species is used for its anti-inflammatory properties [37], and the aerial parts are used for their strong antioxidant properties [38]. The dried aerial parts of this taxon are more or less available through the medicinal plant trade at least in Crete [37], while their commercial demand is constantly increasing on the world market [34].

*S. syriaca* subsp. *syriaca* or Malotira as locally called in Crete (Figure 5B) is a local endemic restricted to the high mountain ranges of a single island of Greece (Crete), with wild-growing populations in decline due to over-collection [19]. Therefore, it is considered as Critically Endangered [30]. Malotira has a low yield of essential oil but a great diversity of compounds identified to date in its aerial parts [39]. Monoterpene hydrocarbons represent the majority of compounds (34.2%) and eubotriol was identified for the first time in the aerial parts of this taxon [23]. From the aerial parts of ex-situ propagated and cultivated material, essential oil, phytosterols, ent-kaurene diterpenes, and flavone have been isolated [23]. Research concerning the asexual propagation, the chemical profile of the dichloromethane extract as well as of the essential oil of the plant material propagated and cultivated ex situ of *S. syriaca* subsp. *syriaca* has been carried out recently and the taxon is therefore proposed for sustainable exploitation as new industrial crop [23]. It has already become a local crop MAP in Crete with established value chains and increased usability [22,23], profiting from the trade of other perennial crops of the genus *Sideritis* such as *S. scardica* Griseb. and *S. raeseri* Boiss. & Heldr. subsp. *raeseri*.

Cretan marjoram (*Origanum microphyllum*) is a local endemic plant of Crete (Figure 5C) found mainly in the western part of the island [28]. It is known and appreciated by local people in Crete particularly for its calming and anti-spasmodic properties and pleasant scent. In Crete, it is occasionally traded at local markets in dried form and it is harvested from the wild, and is therefore considered as Critically Endangered [30]. Previous studies suggested the domestication of this NUP as a MAP with promising agro-alimentary potential [22]. In our study, *O. microphyllum* scored above average (59.3%) in the medicinal-cosmetic sector. This was due to the fact that closely related plants such as *O. majorana* are associated with an EMA herbal monograph, and additionally, relevant phytochemical studies showed that its essential oil contains high levels of terpinen-4-ol (25.0%), sabinene (14.7%) and linalool (11.2%), exhibiting antioxidant and antiproliferative activities against two human cancer cell lines, LoVo and HepG2 [40]. Other studies, report that extracts from *O. microphyllum* aerial parts are effective against the parasite *Leishmania donovani* complex which causes visceral leishmaniasis, a common disease in the world including the Mediterranean and Maghreb [41]. The noteworthy medicinal potential of *O. microphyllum* suggest the need of its domestication because this would allow its sustainable development as a potential new herbal medicine. To date there are attempts already undertaken for its pilot propagation and field cultivation in agricultural settings locally in Crete [42].

### 4.2. Top Evaluated NUPs of Mediterranean Coast-Rif of Morocco

**Figure 6:**
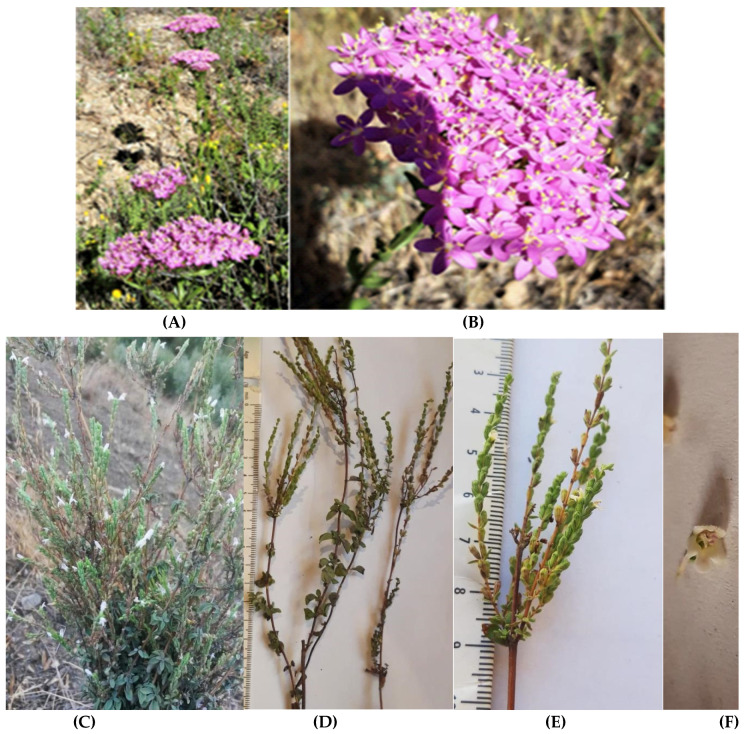

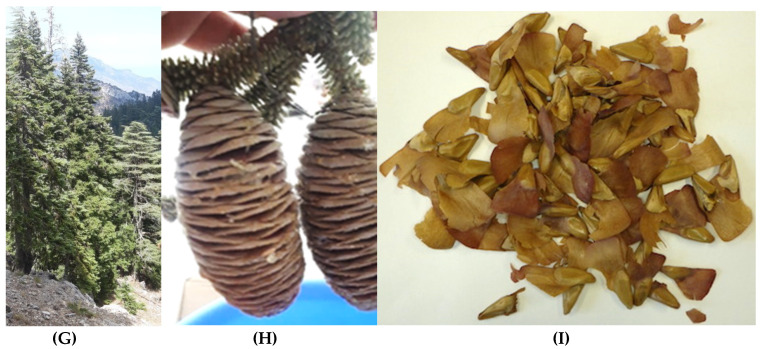
Top-evaluated local endemic plants of Rif-Mediterranean coast of Morocco with interesting medicinal potential: (**A**,**B**): *Centaurium erythraea* subsp. *bifrons* plants in wild habitat (**A**) with impressive inflorescence (**B**); (**C**–**F**): *Origanum elongatum* in its wild habitat (**C**), and flowering shoots (**D**), inflorescence (verticillasters) (**E**), and bilabiate corolla (**F**) of collected sample; (**G**–**I**): *Abies marocana* Trab. individuals (**G**), typical cones (**H**), and winged seeds (**I**).

In Morocco, the local population of Mediterranean Coast-Rif has been a custodian of traditional medicine with long experience in its use [43,44]. However, the 94 local endemic NUPs of this North African region represent unique plant genetic resources for which only scarce information is available about their ethnobotanical or medicinal-cosmetic value. To our knowledge, no medicinal use is reported for the highest evaluated taxon in the case of Morocco, i.e., the non-threatened Moroccan knapweed (*Centaurium erythraea* subsp*. bifrons*) which is a geographical subspecies of an important MAP with approved indications and beneficial health claims. At species level, the common knapweed (or red knapweed, also known as feverfew, Figure 6) is one of the most widely used bitter herbs in the pharmacopoeia of 23 different countries and its inflorescences were one of the multiple constituents of the western maritime pharmacopoeia theriac in the 18th century [45]. The medicinal interest lies in its bitter attributes, which stimulate the secretions of the liver and stomach (difficult digestions, dyspepsia, hepatic insufficiency) while externally, the fresh plant material may be used to treat wounds by local application, while a homeopathic remedy made from *C. erythraea* is also used and marketed in several countries for the treatment of liver and gallbladder disorders [46]. Due to confirmed non-toxicity of the aqueous extract of the species [47], there is a wide margin of safety for the traditional therapeutic use of C*. erythraea* including its subspecies. Most probably this applies also for the Moroccan subsp. *bifrons* and this taxon could provide a potential natural source of bioactive compounds with diverse benefits to human health as evidenced at the species level. Comparative phytochemical analyses (composition and yield) of infraspecific taxa of *C. erythraea* could determine the degree of efficiency of each subspecies. This could specify and confirm the uses for the specific subspecies focused herein, and may allow the development of targeted research for high added-value products in the medicinal or other sectors and their coordinated communication and exclusive marketing in the frame of a sustainable exploitation strategy. Such attempts could facilitate conservation perspectives for the subspecies in concern, bringing also benefits both for local communities and domestic economic development.

*Origanum elongatum* (Bonnet) Emb. & Maire (Lamiaceae) is an interesting non-threatened MAP species which is used to treat several diseases, and exhibits various biological activities such as antibacterial, antifungal, antiparasitic, antiviral, antioxidant, vasodilator, corrosion inhibitor, and hepatoprotective effects [48]. In addition, the essential oil of this species could preserve nutritional and organoleptic qualities, and is therefore suitable for food and culinary preparations [49]. The toxicological investigations revealed the safety of use for *O. elongatum* as well as the hepatoprotection of its methanol extract against hepatotoxic products or drugs [50]. With regards to ex-situ conservation and sustainable exploitation, the high percentage of seed germination trials achieving 88% [51] could be easily used to bridge gaps propagation-wise, which in turn allows for its sustainable cultivation in pilot scales, and may facilitate the introduction of a new crop for essential oil extraction, thus leading to possible subsequent commercialization.

The Moroccan fir (*Abies marocana* Trab.) is a relict tree (Figure 6) confined to the Rif region of Morocco with a very small area of occupancy (estimated at 28 km^2^), coupled with decreasing population trend. It is therefore classified according to IUCN (International Union for the Conservation of Nature) criteria as Endangered [52]. A study conducted by the Moroccan Ministry of Agriculture resulted in the creation of the 60,000 ha Talassemtane natural park to protect the remaining areas of the Moroccan fir, and in 2006 the Talassemtane natural park was included in the first intercontinental Mediterranean biosphere reserve of UNESCO [53]. The seeds’ essential oil of the Moroccan fir has been extensively used in Moroccan folk medicine to treat respiratory disorders [54]. The high proportion of limonene recently discovered in its oil reveals a potential interest in the perfume industry [55]. Although a variety of diterpenoids, cadinans, and cholestanes have been identified in *A. marocana* [56], these data remain insufficient, and therefore targeted phytochemical investigations are needed. Research carried out for 19 *Abies* taxa has identified to date 277 compounds (mainly terpenoids, flavonoids, lignans, phenols, and steroids) several of which fall within the composition of certain cosmetic marketed products [56]. In the same line, 90 constituents have been isolated and identified from seeds and cone scales of *A. alba* Mill., which makes these organs a potential interesting source of molecules for the perfume industry [57]. Similar investigations are necessary to reveal the potentially valuable diversity of molecules and bioactive compounds with cosmetic or medicinal potential produced naturally by *A. marocana*. The multifaceted evaluations carried out by Krigas et al. [8] and Libiad et al. [22] also revealed an interesting potential of the Moroccan fir in the ornamental and agro-alimentary sectors. However, the economic benefits of this taxon can only be achieved through its domestication, since previous work has already reported a decline in its natural population as well as poor natural regeneration [58,59]. To this end, new attempts are currently being undertaken to facilitate its seed propagation for both conservation purposes and sustainable exploitation strategies in the benefit of local communities and domestic development [60].

### 4.3. Top-Evaluated NUPs of Tunisia

**Figure 7:**
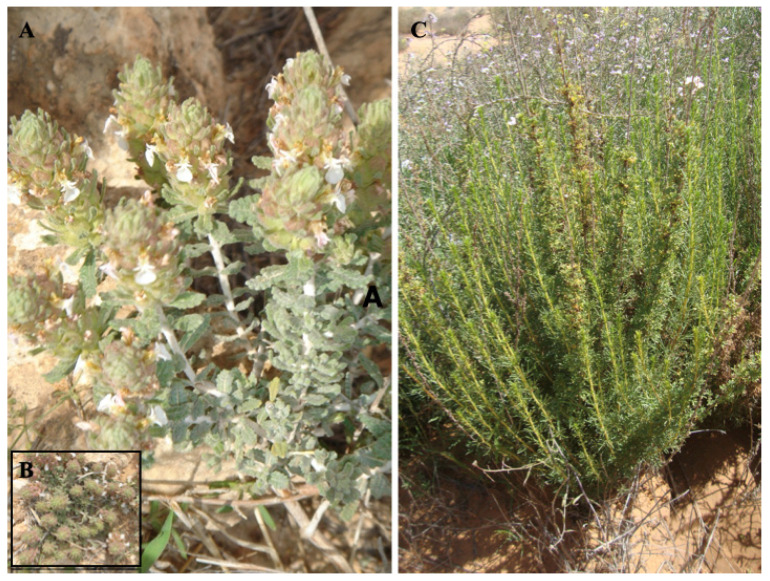
Top-evaluated local endemic plants of Tunisia with strong medicinal-cosmetic potential: (**A**) Flowering shoots with inflorescences (verticillasters) of *Teucrium alopecurus* and taxon’s habit (**B**); (**C**) Wild-growing plant individual of *Artemisia campestris* subsp. *cinerea*.

Extensive studies exemplified by *T. polium* L. show a wide range of therapeutic properties such as antioxidants, anticancer, anti-inflammatory, hypoglycemic, hepatoprotective, lipid-lowering, antibacterial, and antifungal [61]. The ethnobotanical use of some of the Tunisian NUPs with interesting medicinal-cosmetic potential such as the non-threatened *Teucrium alopecurus* (Figure 7A,B) and *Artemisia campestris* subsp. *cinerea* (Figure 7C) is very old [62,63,64], and this fact has been recently confirmed during ethnobotanical surveys carried out from 2014 to 2019 [65]. Fifty-seven compounds have been identified in the essential oil of the Tunisian fox tail germander *T. alopecurus* [66] with sesquiterpenes as major constituent (61.3%). Phytosterols, in particular beta-sitosterol and tocopherols, have been identified in *T. alopecurus* seed oil which have been considered as important lipophilic constituents of seed oils due to the multiple beneficial effects and ability to prevent certain chronic diseases [67,68,69], confirming the importance of this specific oil for pharmaceutical use. Moreover, the essential oil of the endemic Tunisian *T. sauvagei* shows antioxidant and antifungal activities [70]. These facts may render these local endemic *Teucrium* spp. of Tunisia as important MAPs which should not be considered as NUPs anymore, thus deserving a sustainable exploitation strategy at local scales and beyond. It is known that the members of the genus *Teucrium* have long been appreciated for their beneficial properties in traditional medicine [61,62,63,64,65,66,67,68,69,70]. However, further studies are needed at species-specific level to identify the active compounds and to verify or discover new relevant pharmacological activities of local endemic NUPs in this genus, such as the two Cretan endemics (*T. alpestre* Sm. and *T. cuneifolium* Sm., [19]), the 8 taxa restricted to the Mediterranean coast-Rif of Morocco, i.e., *T. afrum* (Emb. & Maire) Pau & Font Quer subsp. *rubriflorum* (Font Quer & Pau) Castrov. & Bayon, *T. chlorostachyum* Pau & Font Quer subsp. *chlorostachyum* and subsp. *melillense* (Maire & Sennen) El Oualidi, Mathez & T. Navarro, *T. grosii Pau*, *T. gypsophilum* Emb. & Maire, *T. huotii* Emb. & Maire, *T. rifanum* (Maire & Sennen) T. Navarro & El Oualidi and *T. rotundifolium* Schreb. subsp. s*anguisorbifolium* (Pau & Font Quer) E. Cohen [21], as well as the 6 Tunisian endemic taxa, i.e., *T. alopecurus Noe* (Figure 6A,B), *Teucrium luteum* (Mill.) Degen subsp. *gabesianum* (S. Puech) Greuter, *T. nablii* S. Puech, *T. radicans* Bonnet & Barratte, *T. sauvagei* Le Houér., and *T. schoenenbergeri* Nabli [21].

Available ethnobotanical data indicate that *Artemisia campestris* subsp. *cinera* (Figure 6C) holds important place in the Maghreb traditional medicine due to its effectiveness against scorpion and ophidian envenomations [62,63,64,65] which constitute a serious health problem, particularly in the small rural villages of south-eastern Tunisia, situated far from hospitals. Recent phytochemical studies have confirmed the antivenom activity of *A. campestris* against the venoms of the scorpion *Androctonus australis garzonii,* and the viper *Macrovipera lebetina* [71,72]. Furthermore, this taxon exhibits a wide range of biological properties such as antioxidant and antimicrobial properties [73,74], as well as antidiabetic, antihyperlipidemic, anti-inflammatory, and antihypertensive properties [75]. *A. campestris* subsp. *cinerea* could also be a promising source for ongoing research on leishmanicidal compounds of plant origin carried out worldwide and particularly in Tunisia [53,76], given the high toxicity of the available chemical drug [76]. Indeed, phytochemical studies have demonstrated the hypothesis of a plausible contribution of the flavonoids of *A. campestris* (luteolin, quercetin, erodictyol, taxifolin, and sakuranetin) in the inhibition of the emergence and the development of different forms of leishmaniasis [75]. With no domestic sustainable exploitation strategy for this taxon allowing for broader documentation and wider transnational usability, the natural effectiveness of this taxon against scorpion and ophidian envenomations [62,63,64,65] will be geographically compromised and will only be used at local scales, despite the fact that envenomations represent a widespread serious health situation affecting many people living in small rural villages far from hospitals in Northern Africa and elsewhere.

## 5. Conclusions

The Mediterranean Basin hosts a great diversity of plants with medicinal potential, a large part of which still remains neglected and underutilized. This particularly applies to range-restricted plants confined to specific geographical areas (local endemic NUPs) despite the fact that such unique floristic elements could provide considerable and profitable medical value for local communities. However, any attempt for exploitation of these phytogenetic resources should be implemented in a sustainable way, ensuring MAP species survival and habitat conservation. Any traditional knowledge or newly discovered knowledge associated with MAPs in decline or threatened with extinction at local and global scales should be prioritized and can be effective in the conservation of range-restricted biological resources. However, any conservation attempt targeted in this direction is difficult to manage or to succeed when introduced and promoted by outside experts. Therefore, to conserve the diversity of local endemic phytogenetic resources used by indigenous communities, it is imperative to involve and engage domestic groups of people in conserving them locally, as they already know or they should apparently be aware of how the different interaction factors work with each other to imperil their sustainability and survival. On the other hand, traditional ethnobotanical uses of local endemic MAPs deserve to be further documented and prioritized, encouraging investigations with clear focus on MAPs that are neither abundant nor widespread but are range-restricted in small geographical areas and are used by local ethnic communities.

In this study, a co-creative and multidisciplinary approach has been developed involving participatory procedures and consensus on evaluation parameters and rules for the multifaceted medicinal-cosmetic evaluation of NUPs. Upon application of the new methodology, comprehensive assessments were generated regarding 399 unique floristic elements (single-region or single-country endemic plants) of three Mediterranean regions (Crete, Greece; Mediterranean coast-Rif of Morocco; Tunisia). As an outcome of this study, hierarchical rankings were produced regarding the detected medicinal-cosmetic potential. These are discussed in terms of feasibility and readiness timescale assessments for the sustainable exploitation of the local endemic NUPs. Species-wise, eight cases were outlined: *Origanum dictamnus*, *Sideritis syriaca* subsp*. syriaca* *and Origanum microphyllum* were indicated as the most prominent ones, followed by three North Moroccan taxa (*Centaurium erythraea* subsp. *bifrons*, *Origanum elongatum, Abies marocana*), and two Tunisian endemic NUPs (*Artemisia campestris* subsp. *cinerea*, *Teucrium alopecuros*).

Although more research work is necessary to unlock the full potential of the evaluated herein local endemic NUPs, this study showed that several wild-growing local endemic plants of Mediterranean regions have emerged with interesting medicinal-cosmetic potential (30 taxa). These NUPs may serve in the future as new functional foods, cosmetics, antidotes and/or nutraceuticals and may prove to offer new possibilities for local development, if appropriately managed and conserved at local scales. This study outlined the urgent need to develop the domestication procedure for range-restricted species of medicinal-cosmetic interest, focusing on propagation and cultivation trials as a means to facilitate both ex-situ conservation and future sustainable exploitation strategies, thus promoting wider usability of local endemic with significant potential and/or usability of closely related MAPs of different regions with probably similar medicinal potential. To this end, conservation of threatened phytogenetic resources both in-situ and ex-situ is a top priority for local communities and stakeholders which enables at the same time the sustainable utilization of phytogenetic resources.

## Figures and Tables

**Table 1:** Sector-specific attributes and score values designated for the evaluation of the medicinal-cosmetic potential (Level I evaluation, L-I) of the local endemic plants of Crete (Greece), Mediterranean coast-Rif of Morocco, and Tunisia, outlining the escalation of interest and the directionality of scoring. For examples on scoring of the studied taxa, guidelines and data sources used, see Supplementary Material S1.

ATTRIBUTE	Short Description	SCORE 0	SCORE 1	SCORE 2	SCORE 3	SCORE 4	SCORE 5	SCORE 6	Possible Scores
Superfood potential	Evidence-based ‘superfood’ characterization	NO	Uncertain/ Ambiguous	-	Under investigation	Possible	-	YES	0, 1, 3, 4, 6
Identified ethnobotanical uses	Documented ethnobotanical value	NO	-	-	Under investigation	-	Possible	YES	0, 3, 5, 6
Medicinal potential	Documented medicinal value	No data	-	-	Under investigation	Possible	-	YES	0, 3, 4, 6
Distinct ethnobotanical uses	Number of documented ethnobotanical uses	No data	1	-	2 or 4	-	-	5 or more	0, 1, 3, 6
Distinct medicinal properties	Number of documented medicinal properties	No data	1	2 or 3	4 or 5	6 or 7	8 or 9	10 or more	0–6
EMA Monograph status	Preparation or publication status of the herbal monograph of the European Medicines Agency (EMA)	No monograph	Assigned	Initiated	Under preparation	Published draft	Under review	Finalized	0–6
Approved indications	Number of distinct indications approved by the EMA	No approved	1	2	3	4	5	6 or more	0–6
Identified phytochemical compounds	Studied categories of phytochemicals and concomitant biological activities	Not studied	-	-	Preliminary chemical profile	General chemical profile	-	Analytical chemical profile	0, 3, 4, 6
Poisonousness- Toxicity	Presence of toxic or poisonous compounds	Toxic/ poisonous	-	Possible	Suspected	Uncertain/ Ambiguous	-	Not toxic/not poisonous	0, 2, 3, 4, 6

## Data Availability

The data presented in this study are available on request from the corresponding authors.

## Supplementary Materials

The following are available online at https://www.mdpi.com/article/10.3390/biology10121344/s1, Supplementary Material S1: Level I evaluation examples with explanations, guidelines, data sources used and scoring per attribute assessing the medicinal-cosmetic potential of the local endemic plants of Crete, Greece (223 taxa), Mediterranean coast-Rif of Morocco (94 taxa), and Tunisia (82 taxa), Supplementary Material S2: Hierarchically arranged percentages (%) of the maximum possible scores achieved regarding the medicinal-cosmetic potential of the local endemic plants of Crete, Rif-Mediterranean coast of Morocco, and Tunisia (Level I evaluation) outlining blue cell cases (Highest: >70%), green cell cases (above average to high: 55–70%), yellow cell cases (above average: 50–55%), grey cell cases (below average to low: >35–50%) and no color cases (lower: <35%). For each taxon score values regarding feasibility for sustainable exploitation (Level II evaluation) and characterization about readiness timescale for value chain creation (Level III) are retrieved from (Krigas et al., 2021 [8]).
