# Supplementary material for: Medicinal-Cosmetic Potential of the Local Endemic Plants of Crete (Greece), Northern Morocco and Tunisia: Priorities for Conservation and Sustainable Exploitation of Neglected and Underutilized Phytogenetic Resources
Source: Biology (Basel). 2021 Dec 16;10(12):1344. doi: 10.3390/biology10121344 (PMC8698715; doi:10.3390/biology10121344)
Supplement: Supplementary file 1 [file biology-10-01344-s001.zip › biology-1497607-supplementary/Supplementary Material S1.pdf]

Supplementary Material S1: Level I evaluation examples with explanations, guidelines, data sources used and scoring per attribute assessing the medicinal-cosmetic potential of the local endemic plants of Crete, Greece (223 taxa), Mediterranean coast-Rif of Morocco (94 taxa) and Tunisia (82 taxa)

Level I – Evaluation of the potential in the medicinal-cosmetic sector

Attribute 1: Super food potential (Evidence-based 'superfood' characterization)
Guidelines: Review of pharmacognostic literature and enthnobotanical data; additionally, consultation with experts and/or interviews with elderly people / Yes = extant documentation of nutritional value and health promoting agents; Possible = if at least some evidence is available; Under investigation = studies are in progress; No = uncertain/ambiguous data.
Data sources: Crete - Stikoudi et al. 2016, Maietta et al. 2018; additionally, best expert judgment.
Score 6: Taxa suitable as super foods, e.g., *Origanum dictamnus* (EMA 2013, Krigas et al. 2015, Stikoudi et al. 2016, Maietta et al. 2018) and *Sideritis syriaca* subsp. *syriaca* (Stikoudi et al. 2016, EMA 2016). **Score 4:** Taxa potentially suitable as super foods, e.g., *Calamintha cretica*. **Score 3:** Taxa with suspected super food value, e.g., *Helichrysum heldreichii*. **Score 1:** Taxa with uncertain super food value (no case). **Score 0:** Taxa without documentation or cases with no estimation achieved, e.g., *Carlina diae*.

Attribute 2: Identified ethnobotanical uses (Documented ethnobotanical value)
Guidelines: Review pharmacognostic literature and enthnobotanical data; additionally, consultation with experts and/or interviews with elderly people / Yes = documented uses; Possible = identified uses only in congeners (other species of the same genus); Under investigation = studies in progress; No = No data
Data sources: Crete - Axiotis et al. 2018, EMA 2013, 2016, Gonzalez- Tejero et al. 2008, Hadjichambis et al. 2009, Heinrich et al. 2006, Heywood & Skoula 1999, Pieroni et al. 2006, Tsioutsiou et al. 2019, Leonidiou 2016, Psaroudaki 2012; Morocco - Khabbach et al. 2011, 2012; additionally, ethnobotanical survey and best expert judgment.
Score 6: Taxa with documented ethnobotanical uses, e.g., *Campanula pelviformis* (Psaroudaki 2012) and *Origanum elongatum* (ethnobotanical survey). **Score 5:** Taxa with potential ethnobotanical uses, e.g., *Centaurea redempta* subsp. *redempta* (Leonidiou 2016). **Score 3:** Taxa with suspected ethnobotanical uses (no case for Crete). **Score 0:** Taxa without documentation, e.g., *Filago wagenitziana*.

Attribute 3: Medicinal potential (Documented medicinal value)
Guidelines: Review of pharmacognostic literature and/or enthnobotanical data; additionally, consultation with experts and/or interviews with elderly people / Yes = identified and documented properties; Possible = identified properties only in congeners (other species of the same genus); Under investigation = studies in progress; No = No data or unknown properties
Data sources: Crete - Axiotis et al. 2018, EMA 2013, 2016, Gonzalez-Tejero et al. 2008, Hadjichambis et al. 2009, Heinrich et al. 2006, Heywood & Skoula 1999, Krigas et al.

2015, Pieroni et al. 2006, Leonidiou 2016, Stikoudi et al. 2016, Tsioutsiou et al. 2019, Psaroudaki 2012; additionally, best expert judgment.
Score 6: Taxa with documented medicinal uses, e.g., *Origanum dictamnus* (EMA 2013, Krigas et al. 2015, Stikoudi et al. 2016, Maietta et al. 2018) and *Sideritis syriaca* subsp. *syriaca* (EMA 2016, Stikoudi et al. 2016). **Score 4:** Taxa with potential medicinal uses, e.g., *Viola alba* subsp. *cretica*. **Score 3:** Taxa with suspected medicinal uses, e.g., *Micromeria hispida*. **Score 0:** Taxa without documentation, e.g., *Ornithogalum insulare*.

Attribute 4: Distinct ethnobotanical uses (Number of documented ethnobotanical uses)
Guidelines: Review of pharmacognostic literature and/or enthnobotanical data; additionally, consultation with experts and/or interviews with elderly people
Data sources: Crete - Axiotis et al. 2018, Gonzalez- Tejero et al. 2008, Hadjichambis et al. 2009, Heinrich et al. 2006, Heywood & Skoula 1999, Pieroni et al. 2006, Stikoudi et al. 2016, Tsioutsiou et al. 2019, Kloukina et al. 2020, Leonidiou 2016, Psaroudaki 2012; additionally, best expert judgment.
Score 6: Taxa with >4 ethnobotanical uses, e.g., *Origanum dictamnus* and *Sideritis syriaca* subsp. *syriaca* (infusion, decoction, skin and/or hair treatments, natural cosmetics, various culinary preparations, Krigas et al. 2015, Stikoudi et al. 2016, Maietta et al. 2018, Kloukina et al. 2020). **Score 3:** Taxa with 2-4 ethnobotanical uses, e.g., *Origanum microphyllum* (infusion, decoction, culinary preparations). **Score 1:** Taxa with one ethnobotanical use, e.g., *Campanula pelviformis* (wild edible green, Psaroudaki 2012). **Score 0:** Taxa without data on their ethnobotanical uses, e.g., *Cephalanthera cucullata*.

Attribute 5: Distinct medicinal properties (Number of documented medicinal properties)
Guidelines: Review of pharmacognostic literature and/or enthnobotanical data; additionally, consultation with experts and/or interviews with elderly people
Data sources: Crete - Leonidiou 2016, Stikoudi et al. 2016 ; Morocco- Balahbib et al., 2021)
Score 6: Taxa with >9 medicinal properties, e.g., *Origanum dictamnus* (against cold, against infections, antioxidant, in ammenorea and gastrointestinal disorders, antihelminthic, for neurological disorders, anticancer, antihemorroid, antidysenterical, against lethargy; Krigas et al. 2015, Leonidiou 2016, Stikoudi et al. 2016, Maietta et al. 2018). **Score 5:** Taxa with 8-9 medicinal properties, e.g. *Origanum elongatum*(such as antibacterial, antifungal, antiparasitic, antiviral, antioxidant, vasodilator, corrosion inhibitor, and hepatoprotective effects; Balahbib et al., 2021) . **Score 4:** Taxa with 6-7 medicinal properties (no case). **Score 3:** Taxa with 4-5 medicinal properties (no case). **Score 2:** Taxa with 3 medicinal properties, e.g., *Origanum microphyllum* (infection, oxidation and cancer, Leonidiou 2016). **Score 1:** Taxa with 2 medicinal properties, e.g., *Onopordum bracteatum* subsp. *creticum* (gastrointestinal disorders and nephrolithiasis, Leonidiou 2016). **Score 0:** Taxa without known medicinal properties, e.g., *Cotoneaster creticus*.

Attribute 6: EMA Monograph (Publication status of the herbal monograph of the European Medicines Agency)
Guidelines: Consult EMA monographs' publication status (ema.europa.eu/en)

Data sources: European Medicines Agency (www.ema.europa.eu/)
Score 6: Taxa with finalized monograph, e.g., *Sideritis syriaca* subsp. *syriaca* (EMA 2016) & *Origanum dictamnus* (EMA 2013). **Score 5:** Taxa with monograph under review (no case). **Score 4:** Taxa with published draft monograph (no case). **Score 3:** Taxa with under preparation monograph (no case). **Score 2:** Taxa with initiate monograph (no case). **Score 1:** Taxa with assigned monograph (no case). **Score 0:** Taxa without monograph, such as the rest of the Cretan taxa as well as all studied Moroccan (Mediterranean coast-Rif) and Tunisian taxa.

Attribute 7: Approved indications (Number of approved EMA indications)
Guidelines: Consult EMA monographs' publication status (ema.europa.eu/en)
Data sources: European Medicines Agency (www.ema.europa.eu/)
Score 6: Taxa with >5 approved EMA indications (no case). **Score 5:** Taxa with 5 indications (no case). **Score 4:** Taxa with 4 indications (no case). **Score 3:** Taxa with 3 indications, e.g., *Origanum dictamnus* (traditional herbal medicinal product used for the relief of cough associated with cold, mild gastrointestinal disorders and minor skin inflammations and bruises, EMA 2013). **Score 2:** Taxa with 2 indications, e.g., *Sideritis syriaca* subsp. *syriaca* (traditional herbal medicinal product used for the relief of cough associated with cold and mild gastrointestinal discomfort, EMA 2016). **Score 1:** Taxa with 1 indication (no case). **Score 0:** Taxa without indications, such as the rest of the Cretan taxa as well as all studied Moroccan (Mediterranean coast-Rif) and Tunisian taxa.

Attribute 8: Identified phytochemical compounds (studied categories of phytochemicals and concomitant biological activities)
Guidelines: Species-specific literature survey (databases and grey literature) / Preliminary = only studied composition of one category of phytochemicals; General = studied composition in >1 categories of phytochemicals; Analytical = studied composition in >1 categories of phytochemicals accompanied with tested biological activities
Data sources: Crete - Alfaro et al. 2014, Athanasas et al. 2004, Contandriopoulos & Lanzalavi 1968, Demetzos et al. 2000, EMA 2013, 2016, Fokialakis et al. 2007, Krigas et al. 2015, Michalakea et al. 2019, Mitrocotsa et al. 1999, Skoula & Grayer 2004, Stikoudi et al. 2016, Syros et al. 2003. ; Morocco- Balahbib et al., 2021)
Score 6: Taxa with analytical chemical profile, e.g., *Origanum microphyllum and Origanum elongatum*. **Score 4:** Taxa with general chemical profile, e.g., *Teucrium cuneifolium*. **Score 3:** Taxa with preliminary chemical profile, e.g., *Calamintha cretica*. **Score 0:** Taxa not studied yet (no documentation), e.g., *Bupleurum gaudianum*.

Attribute 9: Poisonousness-Toxicity (Presence of dangerous compounds)
Guidelines: Toxic/poisonous = documented toxicity according to literature or after interviews; Possible = toxicity in congeners (other species of the same genus); Suspected = some indications extant; Uncertain-ambiguous = controversy of data; Not toxic / not poisonous = documented absence of toxicity
Data sources: Plants for a Future (www.pfaf.org/); additionally, best expert judgment based on empirical knowledge.

Score 6: Taxa without toxic or poisonous properties, e.g., *Crepis sibthorpiana*. **Score 4:** Taxa with uncertain- ambitious data on their toxicity, e.g., *Bellevalia sitiaca*. **Score 3:** Taxa's toxicity under investigation, e.g., *Hypericum amblycalyx*. **Score 2:** Taxa with potentially toxic or poisonous properties, e.g., *Ranunculus radinotrichus*. **Score 0:** Taxa with documented toxic or poisonous properties, e.g., *Securigera globosa*.

References

Alfaro, R.A.D.; Gomez-Sandoval, Z.; Mammino, L. Evaluation of the antiradical activity of hyperjovinol-A utilizing donor-acceptor maps. *J. Mol. Model.* **2014**, *20, 2337.*

Athanasas, K.; Magiatis, P.; Fokialakis, N.; Skaltsounis, A.L.; Pratsinis, H.; Kletsas, D. Hyperjovinols A and B: Two new phloroglucinol derivatives from *Hypericum jovis* with antioxidant activity in cell cultures. *J. Nat. Prod.* **2004**, *67, 973-977.*

Axiotis, E.; Halabalaki, M.; Skaltsounis, L.A. An ethnobotanical study of medicinal plants in the Greek islands of north Aegean region. *Front. Pharmacol.* **2018**, *9, 409.*

Balahbib, A.; El Menyiy, N.; El Omari, N.; Benali, T.; Guaouguaou, F.-E.; Salhi, N.; Naceiri Mrabti, H.; Bouyahya, A. Phytochemistry, Toxicology, and Pharmacological Properties of *Origanum elongatum. Evid.-Based Complement. Altern. Med.* **2021**, 6658593.

Contandriopoulos, J.; Lanzalavi, M. Contribution À L'étude cytotaxinomique deshypericumde Grèce. *Bull. Soc. Bot. France*, **1968**, *115,* 5-13.

Demetzos, C.; Perdetzoglou, D.; Gazouli, M.; Tan, K.; Economakis, C. Chemical analysis and antimicrobial studies on three species of *Ferulago* from Greece. *Planta Med.* **2000,** *66,* 560-563.

EMA. Final assessment report on *Origanum dictamnus* L., herba (EMA/HMPC/200431/2012); European Medicines Agency/Committee on Herbal Medicinal Products: London, UK, **2013**. Available online: http://www.ema.europa.eu/docs/en_GB/document_library/Herbal_-_HMPC_assessment_report/2013/08/WC500147179.pdf. (accessed on 11 November 2021).

EMA/HMPC. Final Assessment Report on *Sideritis scardica* Griseb.; *Sideritis clandestina* (Bory & Chaub.) Hayek; *Sideritis raeseri* Boiss. & Heldr.; *Sideritis syriaca* L., *Herba* (39455/2015); European Medicines Agency/Committee on Herbal Medicinal Products: London, UK, **2016**; Available online: https://www.ema.europa.eu/en/documents/herbal-report/final-assessment-report-sideritis-scardica-griseb-sideritis-clandestina-bory-chaub-hayek-sideritis_en.pdf (accessed on 1 October 2021).

European Medicines Agency. https://www.ema.europa.eu (Accessed on November 11, 2021).

Fokialakis, N.; Kalpoutzakis, E.; Tekwani, B.L.; Khan, S.I.; Kobaisy, M.; Skaltsounis, A.L.; Duke, S.O. Evaluation of the antimalarial and antileishmanial activity of plants from the Greek island of Crete. *J. Nat. Med.* **2007**, 61, 38-45.

Fournaraki, C. Conservation of threatened plants of Crete – Seed ecology, operation and management of a gene bank. PhD Thesis, National and Kapodistrian University of Athens, Faculty of Biology, Department of Botany, 2010 [In Greek with English abstract].

González-Tejero, M.R.; Casares-Porcel, M.; Sánchez-Rojas, C.P.; Ramiro-Gutiérrez, J.M.; Molero-Mesa, J.; Pieroni, A.; Giusti, M.E.; Censorii, E.; De Pasquale, C.; Della, A.; Paraskeva-Hadjichambi, D.; Hadjichampish, A.; Houmani, Z.; El-Demerdash, M.; ElZayat, M.; Hmamouchi, M.; Eljohrig, S., Medicinal plants in the Mediterranean area: Synthesis of the results of the project Rubia. *J. Ethnopharmacol*. **2008**, *116*, 341-357.

Hadjichambis, A.CH., Paraskeva-Hadjichambi, D.; Della, A.; Giusti, M.E.; De Pasquale, C.; Lenzarini, C.; Censorii, E.; González-Tejero, M.R.; Sánchez-Rojas, C.P.; Ramiro-Gutiérrez, J.M.; Skoula, M.; Johnson, C.; Sarpaki, A.; Hmamouchi, M.; Jorhi, S.; El-Demerdash, M.; El-Zayat, M.; Pieroni, A. Wild and semi-domesticated food plant consumption in seven circum-Mediterranean areas. *Int. J. Food Sci. Nutr*. **2009**, *59*, 383-414.

Heinrich, M.; Müller, W.E.; Galli, C. Local Mediterranean food plants and nutraceuticals. *Forum Nutr. Basel, Karger* **2006**, *59*, 1-17.

Heywood, V.; Skoula, M. The MEDUSA Network: Conservation and sustainable use of wild plants of the Mediterranean Region. In Janick, J., ed., Perspectives on new crops and new uses, VA: ASHS Press, pp. 148-151. 1999.

Kloukina, C.; Tomou, E.M.; Krigas, N.; Sarropoulou, V.; Madesis, P.; Maloupa, E.; Skaltsa, H. Non-polar secondary metabolites and essential oil of *ex situ* propagated and cultivated *Sideritis syriaca* L. subsp. *syriaca* (Lamiaceae) with consolidated identity (DNA Barcoding): towards a potential new industrial crop. *Ind. Crop. Prod*. **2020**, *158*, 112957

Krigas, N.; Lazari, D.; Maloupa, E.; Stikoudi, M. Introducing Dittany of Crete (*Origanum dictamnus* L.) to gastronomy: A new culinary concept for a traditionally used medicinal plant. *Int. J. Gastron. Food Sci.* **2015**, *2, 112-118*.

Leonidiou, F. Medicinal and aromatic plants of Greece: Inventory and conservation priorities. Master Thesis, Faculty of Crop Science, Agricultural University of Athens, 2016 [In Greek with English abstract].

Maietta, M.; Colombo, R.; Corana, F; Papetti, A., Cretan tea (*Origanum dictamnus* L.) as a functional beverage: an investigation on antiglycative and carbonyl trapping activities. *Food Funct.* **2018**, *9(3), 1545-1556*.

Michalakea, E.; Graikou, K.; Aligiannis, N.; Panoutsopoulos, G.; Kalpoutzakis, E.; Roussakis, C.; Chinoua, A. Isolation and structure elucidation of secondary metabolites of two Greek endemic *Inula* species, Biological activities. *Phytochem. Letts*. **2019**, *31,* 155-160.

Mitrocotsa, D.; Skaltsounis, A.L.; Mitaku, S.; Harvala, C.; Tillequin, F. Flavonoid and terpene glycosides from European *Ebenus* species. *Biochem. Syst. Ecol*. **1999,** *27,* 305-307.

Pieroni, A.; Giusti, M.A.; Pesquale, C.; Lenzarini, C.; Censorii, E.; Gonzales-Tejero, M.R.; Sánchez-Rojas, Ch.; Skoula, M.; Johnson, Ch.; Sarpaki, A.; Della, A.; ParaskevaHadijchambi, D.; Hadjichambis, A.Ch.; Hmamouchi, M.; Mohamed El-Demerdash, M.; El-Zayat, M.; Houmani, Z.; Scherazed, M. Circum-Mediterranean cultural heritage and medicinal plant uses in traditional animal health care: a field survey in eight selected areas within the RUBIA project. *J. Ethnobiol. Ethnomed*. **2006,** *2*, 16.

Plants for a Future. https://www.pfaf.org (accessed on 11 November 2021)

Psaroudaki, A. Recording, botanical identification, genetic diversity, attributes of indigenous edible plants of Crete and their participation in the current nutritional pattern. Ph. D. Thesis, Faculty of Crop Science, Agricultural University of Athens, 2012 [In Greek with English abstract].

Skoula, M.; Grayer, R.J.; Kite, G.C. Surface flavonoids in *Coridothymus capitatus* and *Thymbra calostachya* (Lamiaceae). *Biochem. Syst. Ecol.* **2004,** *32*, 1197-1200.

Stikoudi, M.; Maloupa, E.; Lazari, D.; Krigas, N. Aromagarden and Cooking for Wellness: Discovering secrets of Mediterranean plants. i-Print: Thessaloniki, Greece, 2016**.**

Syros, T.D.; Yupsanis, T.A.; Economou, A.S. Peroxidase activity during rooting of cuttings in *Ebenus cretica* - A preliminary study. *Acta Hortic.* **2003,** *624,* 123-128.

Tsioutsiou, E.E.; Giordani, P.; Hanlidou, E.; Biagi, M.; De Feo, V.; Cornara, L. Ethnobotanical study of medicinal plants used in Central Macedonia, Greece. *Evid. Based Complement. Altern. Med.* **2019**, 4513792.
