# Supplementary material for: Medicinal-Cosmetic Potential of the Local Endemic Plants of Crete (Greece), Northern Morocco and Tunisia: Priorities for Conservation and Sustainable Exploitation of Neglected and Underutilized Phytogenetic Resources
Source: Biology (Basel). 2021 Dec 16;10(12):1344. doi: 10.3390/biology10121344 (PMC8698715; doi:10.3390/biology10121344)
Supplement: Supplementary file 1 [file biology-10-01344-s001.zip › biology-1497607-supplementary/Supplementary Material S2.pdf]

Supplementary Material S2: Hierarchically arranged percentages (%) of the maximum possible scores achieved regarding the medicinal-cosmetic potential of the local endemic plants of Crete, Rif-Mediterranean coast of Morocco and Tunisia (Level I evaluation) outlining blue cell cases (Highest: >70%), green cell cases (above average to high: 55-70%), yellow cell cases (above average: 50-55%), grey cell cases (below average to low: >35-50%) and no colour cases (lower: <35%). For each taxon score values regarding feasibility for sustainable exploitation (Level II evaluation) and characterization about readiness timescale for value chain creation (Level III) are retrieved from (Krigas et al. 2021).

A. Local endemic plants of Crete (Greece)

Taxa	Potential (Level I)	Feasibility (Level II)	Readiness timescale (Level III)
Origanum dictamnus	94.44	91.67	Already achieved
Sideritis syriaca subsp. *syriaca*	81.48	66.67	Short term
Origanum microphyllum	59.26	52.78	Medium term
Thymbra calostachya	48.15	55.56	Short term
Calamintha cretica	46.30	69.44	Short term
Onopordum bracteatum subsp. *creticum*	46.30	38.89	Long term
Phlomis lanata	46.30	51.39	Medium term
Eryngium ternatum	44.44	54.17	Medium term
Teucrium alpestre	40.74	36.11	Long term
Teucrium cuneifolium	40.74	63.89	Short term
Campanula pelviformis	38.89	43.06	Long term
Helichrysum doerfleri	38.89	45.83	Long term
Helichrysum heldreichii	38.89	61.11	Short term
Hypericum amblycalyx	38.89	33.33	Indeterminable
Hypericum jovis	38.89	56.94	Short term
Muscari spreitzenhoferi	37.04	37.5	Long term
Petromarula pinnata	37.04	45.83	Long term
Anchusa cespitosa	35.19	50	Medium term
Campanula cretica	33.33	36.11	Long term
Campanula tubulosa	33.33	33.33	Indeterminable
Centaurea idaea	33.33	36.11	Long term
Medicago arborea subsp. *strasseri*	33.33	50	Medium term
Inula candida subsp. *decalvans*	31.48	44.44	Long term

Arum idaeum	29.63	29.17	Indeterminable
Ferulago thyrsiflora	29.63	37.5	Long term
Polygonum idaeum	29.63	47.22	Long term
Scutellaria sieberi	29.63	36.11	Long term
Viola alba subsp. *cretica*	29.63	23.61	Indeterminable
Viola fragrans	29.63	34.72	Indeterminable
Cynoglossum sphacioticum	27.78	40.28	Long term
Ebenus cretica	27.78	45.83	Long term
Micromeria hispida	27.78	45.83	Long term
Sanguisorba cretica	27.78	38.89	Long term
Verbascum arcturus	27.78	47.22	Long term
Astragalus creticus subsp. *creticus*	25.93	22.22	Indeterminable
Astragalus creticus subsp. *minoicus*	25.93	23.61	Indeterminable
Erysimum creticum	24.07	44.44	Long term
Erysimum mutabile	24.07	29.17	Indeterminable
Erysimum raulinii	24.07	40.28	Long term
Hypericum empetrifolium subsp. *tortuosum*	24.07	27.78	Indeterminable
Allium bourgeaui subsp. *creticum*	22.22	37.5	Long term
Staehelina petiolata	22.22	54.17	Medium term
Allium circinnatum subsp. *circinnatum*	20.37	29.17	Indeterminable
Allium dilatatum	20.37	27.78	Indeterminable
Allium platakisii	20.37	41.67	Long term
Asperula crassula	20.37	48.61	Long term
Asperula idaea	20.37	38.89	Long term
Asperula pubescens	20.37	33.33	Indeterminable
Asperula rigida	20.37	33.33	Indeterminable
Biarum davisii	20.37	41.67	Long term
Biarum tenuifolium subsp. *idomenaeum*	20.37	18.06	Indeterminable
Campanula creutzburgii	20.37	33.33	Indeterminable
Campanula hierapetrae	20.37	58.33	Short term
Campanula jacquinii	20.37	38.89	Long term
Campanula saxatilis subsp. *saxatilis*	20.37	59.72	Short term
Campanula spatulata subsp. *filicaulis*	20.37	23.61	Indeterminable
Carlina diae	20.37	58.33	Short term
Centranthus sieberi	20.37	44.44	Long term
Cerastium brachypetalum subsp. *doerfleri*	20.37	45.83	Long term
Cerastium deschatresii	20.37	23.61	Indeterminable
Cerastium scaposum subsp. *peninsularum*	20.37	26.39	Indeterminable
Cerastium scaposum subsp. *scaposum*	20.37	26.39	Indeterminable

Convolvulus argyrothamnos	20.37	59.72	Short term
Crepis auriculifolia	20.37	58.33	Short term
Crepis sibthorpiana	20.37	52.78	Medium term
Cuscuta atrans	20.37	43.06	Long term
Hieracium schmidtii subsp. *creticum*	20.37	22.22	Indeterminable
Hypochaeris tenuiflora	20.37	33.33	Indeterminable
Lactuca alpestris	20.37	29.17	Indeterminable
Lathyrus neurolobus	20.37	41.67	Long term
Limonium chersonesum	20.37	27.78	Indeterminable
Limonium chrisianum	20.37	27.78	Indeterminable
Limonium cornarianum	20.37	47.22	Long term
Limonium creticum	20.37	52.78	Medium term
Limonium elaphonisicum	20.37	50	Medium term
Limonium grabusae	20.37	27.78	Indeterminable
Limonium hierapetrae	20.37	33.33	Indeterminable
Limonium minoicum	20.37	27.78	Indeterminable
Limonium recticaule	20.37	27.78	Indeterminable
Limonium sougiae	20.37	29.17	Indeterminable
Limonium xerocamposicum	20.37	27.78	Indeterminable
Lomelosia albocincta	20.37	38.89	Long term
Lomelosia minoana subsp. *asterusica*	20.37	50	Medium term
Lomelosia minoana subsp. *minoana*	20.37	40.28	Long term
Lomelosia sphaciotica subsp. *decalvans*	20.37	27.78	Indeterminable
Lomelosia sphaciotica subsp. *sphaciotica*	20.37	27.78	Indeterminable
Micromeria sphaciotica	20.37	40.28	Long term
Ononis verae	20.37	27.78	Indeterminable
Orchis sitiaca	20.37	25	Indeterminable
Orchis spitzelii subsp. *nitidifolia*	20.37	25	Indeterminable
Scorzonera mollis subsp. *idaea*	20.37	45.83	Long term
Silene ammophila subsp. *ammophila*	20.37	52.78	Medium term
Silene antri-jovis	20.37	40.28	Long term
Silene flavescens subsp. *dictaea*	20.37	51.39	Medium term
Silene integripetala subsp. *greuteri*	20.37	37.5	Long term
Silene pinetorum subsp. *pinetorum*	20.37	40.28	Long term
Silene pinetorum subsp. *sphaciotica*	20.37	40.28	Long term
Silene sieberi	20.37	36.11	Long term
Silene variegata	20.37	29.17	Indeterminable
Tamarix minoa	20.37	30.56	Indeterminable
Tragopogon lassithicus	20.37	50	Medium term

Acantholimon androsaceum	16.67	33.33	Indeterminable
Adonis cretica	16.67	22.22	Indeterminable
Anthemis abrotanifolia	16.67	38.89	Long term
Anthemis filicaulis	16.67	33.33	Indeterminable
Anthemis glaberrima	16.67	62.5	Short term
Anthemis samariensis	16.67	33.33	Indeterminable
Anthemis tomentella	16.67	33.33	Indeterminable
Bellevalia brevipedicellata	16.67	62.5	Short term
Bellevalia juliana	16.67	44.44	Long term
Bellevalia sitiaca	16.67	38.89	Long term
Bufonia stricta subsp. *cecconiana*	16.67	23.61	Indeterminable
Centaurea argentea subsp. *chionantha*	16.67	45.83	Long term
Centaurea argentea subsp. *macrothysana*	16.67	33.33	Indeterminable
Centaurea baldaccii	16.67	36.11	Long term
Centaurea lancifolia	16.67	52.78	Medium term
Centaurea poculatoris	16.67	61.11	Short term
Centaurea redempta subsp. *redempta*	16.67	33.33	Indeterminable
Galium extensum	16.67	27.78	Indeterminable
Galium fruticosum	16.67	44.44	Long term
Galium incanum subsp. *creticum*	16.67	44.44	Long term
Galium incrassatum	16.67	38.89	Long term
Galium incurvum	16.67	23.61	Indeterminable
Limonium calliopsium	16.67	47.22	Long term
Linum caespitosum	16.67	27.78	Indeterminable
Linum doerfleri	16.67	38.89	Long term
Phagnalon pygmaeum	16.67	27.78	Indeterminable
Sedum eriocarpum subsp. *spathulifolium*	16.67	22.22	Indeterminable
Serapias cordigera subsp. *cretica*	16.67	19.44	Indeterminable
Alkanna sieberi	14.81	37.5	Long term
Asplenium creticum	14.81	27.78	Indeterminable
Hypericum aciferum	14.81	48.61	Long term
Hypericum kelleri	14.81	48.61	Long term
Hypericum trichocaulon	14.81	29.17	Indeterminable
Ornithogalum dictaeum subsp. *dictaeum*	14.81	33.33	Indeterminable
Ornithogalum insulare	14.81	22.22	Indeterminable
Trifolium phitosianum	14.81	18.06	Indeterminable
Verbascum spinosum	14.81	40.28	Long term
Astragalus dolinicola	12.96	29.17	Indeterminable
Astragalus idaeus	12.96	50	Medium term

Astragalus nummularius subsp. *nummularius*	12.96	27.78	Indeterminable
Crocus oreocreticus	12.96	41.67	Long term
Crocus sieberi	12.96	33.33	Indeterminable
Euphorbia rechingeri	12.96	44.44	Long term
Euphorbia sultan-hassei	12.96	37.5	Long term
Ranunculus cupreus	12.96	38.89	Long term
Ranunculus radinotrichus	12.96	34.72	Indeterminable
Ranunculus veronicae	12.96	41.67	Long term
Senecio fruticulosus	12.96	34.72	Indeterminable
Anacamptis papilionacea subsp. *alibertis*	11.11	22.22	Indeterminable
Arabis cretica	11.11	29.17	Indeterminable
Bupleurum gaudianum	11.11	51.39	Medium term
Bupleurum kakiskalae	11.11	58.33	Short term
Cirsium morinifolium	11.11	23.61	Indeterminable
Cotoneaster creticus	11.11	33.33	Indeterminable
Dianthus fruticosus subsp. *creticus*	11.11	34.72	Indeterminable
Dianthus fruticosus subsp. *sitiacus*	11.11	30.56	Indeterminable
Dianthus juniperinus subsp. *aciphyllus*	11.11	33.33	Indeterminable
Dianthus juniperinus subsp. *bauhinorum*	11.11	51.39	Medium term
Dianthus juniperinus subsp. *heldreichii*	11.11	33.33	Indeterminable
Dianthus juniperinus subsp. *idaeus*	11.11	33.33	Indeterminable
Dianthus juniperinus subsp. *juniperinus*	11.11	33.33	Indeterminable
Dianthus juniperinus subsp. *kavusicus*	11.11	50	Medium term
Dianthus juniperinus subsp. *pulviniformis*	11.11	33.33	Indeterminable
Dianthus sphacioticus	11.11	34.72	Indeterminable
Dianthus xylorrhizus	11.11	58.33	Short term
Draba cretica	11.11	36.11	Long term
Festuca polita subsp. *cretica*	11.11	18.06	Indeterminable
Gagea omalensis	11.11	27.78	Indeterminable
Myosotis solange	11.11	40.28	Long term
Nepeta sphaciotica	11.11	62.5	Short term
Onobrychis sphaciotica	11.11	52.78	Medium term
Prunella cretensis	11.11	34.72	Indeterminable
Scilla nana subsp. *albescens*	11.11	23.61	Indeterminable
Scilla nana subsp. *nana*	11.11	20.83	Indeterminable
Zelkova abelicea	11.11	52.78	Medium term
Colchicum cretense	9.26	29.17	Indeterminable
Cyclamen graecum subsp. *candicum*	9.26	44.44	Long term
Alyssum fragillimum	7.41	44.44	Long term

Alyssum idaeum	7.41	51.39	Medium term
Alyssum lassiticum	7.41	38.89	Long term
Alyssum sphacioticum	7.41	50	Medium term
Bolanthus creutzburgii subsp. *creutzburgii*	7.41	27.78	Indeterminable
Bolanthus creutzburgii subsp. *zaffranii*	7.41	44.44	Long term
Brachypodium sylvaticum subsp. *creticum*	7.41	34.72	Indeterminable
Carex cretica	7.41	25	Indeterminable
Carex idaea	7.41	19.44	Indeterminable
Catapodium borgesii	7.41	27.78	Indeterminable
Cephalanthera cucullata	7.41	43.06	Long term
Chaenorhinum idaeum	7.41	34.72	Indeterminable
Chaerophyllum creticum	7.41	52.78	Medium term
Cherleria wettsteinii	7.41	34.72	Indeterminable
Epipactis cretica	7.41	25	Indeterminable
Filago wagenitziana	7.41	29.17	Indeterminable
Fritillaria messanensis subsp. *sphaciotica*	7.41	18.06	Indeterminable
Geocaryum creticum	7.41	38.89	Long term
Himantoglossum samariense	7.41	18.06	Indeterminable
Horstrissea dolinicola	7.41	45.83	Long term
Klasea cretica	7.41	26.39	Indeterminable
Noccaea cretica	7.41	34.72	Indeterminable
Noccaea zaffranii	7.41	41.67	Long term
Petrorhagia candica	7.41	37.5	Long term
Petrorhagia dianthoides	7.41	47.22	Long term
Prospero battagliae	7.41	15.28	Indeterminable
Prospero depressum	7.41	31.94	Indeterminable
Prospero hierapytnense	7.41	22.22	Indeterminable
Prospero idaeum	7.41	31.94	Indeterminable
Prospero rhadamanthi	7.41	38.89	Long term
Prospero talosii	7.41	33.33	Indeterminable
Ricotia cretica	7.41	34.72	Indeterminable
Sesleria doerfleri	7.41	56.94	Short term
Telephium imperati subsp. *pauciflorum*	7.41	18.06	Indeterminable
Tulipa bakeri	7.41	41.67	Long term
Tulipa cretica	7.41	41.67	Long term
Tulipa doerfleri	7.41	40.28	Long term
Corydalis uniflora	5.56	20.83	Indeterminable
Dactylis glomerata subsp. *rigida*	5.56	37.5	Long term
Scutellaria hirta	5.56	29.17	Indeterminable

Clematis elisabethae-carolae	3.70	44.44	Long term
Cyclamen confusum	0.00	31.94	Indeterminable
Securigera globosa	0.00	43.06	Long term
Vincetoxicum creticum	0.00	38.89	Long term
Viscum album subsp. *creticum*	0.00	16.67	Indeterminable

B. Local endemic plants of Morocco (Rif-Mediterranean coast)

**Taxa**	**Potential (Level I)**	**Feasibility (Level II)**	**Readiness timescale (Level III)**
Centaurium erythraea subsp. *bifrons*	72.22	40.28	Long term
Origanum elongatum	55.55	36.11	Long term
Abies marocana	44.44	43.06	Long term
Malva vidalii	35.19	30.56	Indeterminable
Rumex brachypodus	35.19	18.06	Indeterminable
Centaurium barrelieroides	27.78	22.22	Indeterminable
Marrubium fontianum	27.78	22.22	Indeterminable
Marrubium heterocladum	27.78	23.61	Indeterminable
Salvia interrupta subsp. *paui*	27.78	25.00	Indeterminable
Scrophularia eriocalyx	27.78	22.22	Indeterminable
Scrophularia fontqueri	27.78	25.00	Indeterminable
Sideritis romoi	27.78	16.67	Indeterminable
Astragalus meuselii	25.93	18.06	Indeterminable
Astragalus tetuanensis	25.93	26.39	Indeterminable
Sonchus briquetianus	25.93	22.22	Indeterminable
Sonchus fragilis	25.93	25.00	Indeterminable
Sonchus masguindalii	25.93	36.11	Long term
Vicia cedretorum	25.93	22.22	Indeterminable
Acis tingitana	24.07	31.94	Indeterminable
Stachys fontqueri	22.22	18.06	Indeterminable
Cytisus grandiflorus subsp. *haplophyllus*	20.37	12.50	Indeterminable
Cytisus striatus subsp. *megalanthus*	20.37	9.72	Indeterminable
Echium canum	20.37	22.22	Indeterminable
Ammiopsis daucoides	18.52	8.33	Indeterminable
Anthemis mauritiana subsp. *mauritiana*	18.52	29.17	Indeterminable
Antirrhinum martenii	18.52	30.56	Indeterminable
Armeria alpinifolia	18.52	30.56	Indeterminable
Bromus erectus subsp. microchaetus	18.52	38.89	Long term
Carduus martinezii subsp. *fontqueri*	18.52	22.22	Indeterminable

Centaurea alibeyana	18.52	25.00	Indeterminable
Centaurea monticola subsp. *orthoacantha*	18.52	22.22	Indeterminable
Centaurea oriolii-bolosii	18.52	22.22	Indeterminable
Centaurea xaveri	18.52	25.00	Indeterminable
Convolvulus vidalii	18.52	29.17	Indeterminable
Eryngium caespitiferum	18.52	25.00	Indeterminable
Ferula bolivari	18.52	22.22	Indeterminable
Ferula fontqueri	18.52	13.89	Indeterminable
Fumaria macrosepala subsp. *megasepala*	18.52	27.78	Indeterminable
Fumaria macrosepala subsp. *obscura*	18.52	27.78	Indeterminable
Fumaria ouezzanensis subsp. *ramosa*	18.52	26.39	Indeterminable
Fumaria sepium subsp. *rugosa*	18.52	26.39	Indeterminable
Genista carpetana subsp. *nociva*	18.52	23.61	Indeterminable
Genista osmariensis	18.52	25.00	Indeterminable
Genista oxycedrina	18.52	19.44	Indeterminable
Helianthemum grosii	18.52	30.56	Indeterminable
Herniaria boissieri subsp. *maroccana*	18.52	29.17	Indeterminable
Hieracium riofrioi	18.52	22.22	Indeterminable
Hypericum andjerinum	18.52	25.00	Indeterminable
Iberis contracta subsp. *rifana*	18.52	18.06	Indeterminable
Iberis grosmiquelii	18.52	22.22	Indeterminable
Lathyrus coerulescens	18.52	13.89	Indeterminable
Linum austriacum subsp. *gomaricum*	18.52	22.22	Indeterminable
Matthiola masguindalii	18.52	18.06	Indeterminable
Ononis alopecuroides subsp. *simulata*	18.52	30.56	Indeterminable
Ononis gines-lopezii	18.52	13.89	Indeterminable
Paronychia maroccana	18.52	23.61	Indeterminable
Ptilostemon abylensis	18.52	20.83	Indeterminable
Saxifraga maweana	18.52	23.61	Indeterminable
Saxifraga rigoi subsp. *maroccana*	18.52	30.56	Indeterminable
Saxifraga tricrenata	18.52	31.94	Indeterminable
Saxifraga werneri	18.52	33.33	Indeterminable
Sedum mucizonia subsp. *abylaea*	18.52	18.06	Indeterminable
Silene abietum	18.52	22.22	Indeterminable
Silene lagrangei	18.52	25.00	Indeterminable
Silene rhiphaena	18.52	30.56	Indeterminable
Silene vidaliana	18.52	22.22	Indeterminable
Teucrium afrum subsp. *rubriflorum*	18.52	23.61	Indeterminable
Teucrium chlorostachyum subsp. *chlorostachyum*	18.52	30.56	Indeterminable

Teucrium chlorostachyum subsp. *melillense*	18.52	30.56	Indeterminable
Teucrium grosii	18.52	26.39	Indeterminable
Teucrium gypsophilum	18.52	30.56	Indeterminable
Teucrium huotii	18.52	25.00	Indeterminable
Teucrium rifanum	18.52	29.17	Indeterminable
Teucrium rotundifolium subsp. *sanguisorbifolium*	18.52	19.44	Indeterminable
Trifolium tastetii	18.52	15.28	Indeterminable
Xiphion rutherfordii	18.52	36.11	Long term
Narcissus albimarginatus	12.96	16.67	Indeterminable
Anthyllis vulneraria subsp. *ajmasiana*	11.11	30.56	Indeterminable
Anthyllis vulneraria subsp. *fatmae*	11.11	38.89	Long term
Arabis pubescens subsp. *leucanthemifolia*	11.11	13.89	Indeterminable
Bromopsis maroccana	11.11	30.56	Indeterminable
Cephalaria mauritanica subsp. *rifana*	11.11	20.83	Indeterminable
Fumana fontqueri	11.11	16.67	Indeterminable
Hemicrambe fruticulosa	11.11	29.17	Indeterminable
Leucanthemopsis longipectinata	11.11	13.89	Indeterminable
Mantisalca amberboides	11.11	8.33	Indeterminable
Moehringia glochidisperma	11.11	25.00	Indeterminable
Rhodalsine senneniana	11.11	25.00	Indeterminable
Rhodanthemum hosmariense	11.11	36.11	Long term
Santolina adscensionis	11.11	16.67	Indeterminable
Sideritis arborescens subsp. *maireana*	11.11	16.67	Indeterminable
Festuca embergeri	3.70	8.33	Indeterminable
Festuca fontqueriana	3.70	12.50	Indeterminable
Festuca rifana	3.70	18.06	Indeterminable

C. Local endemic plants of Tunisia

Taxa	Potential (Level I)	Feasibility (Level II)	Readiness timescale (Level III)
Teucrium alopecurus	57.41	27.78	Indeterminable
Teucrium sauvagei	53.70	22.22	Indeterminable
Artemisia campestris subsp. *cinerea*	50.00	50.00	Medium term
Marrubium aschersonii	48.15	26.39	Indeterminable
Calendula suffruticosa subsp. *suffruticosa*	38.89	36.11	Long term
Ferula tunetana	38.89	22.22	Indeterminable
Teucrium luteum subsp. *gabesianum*	35.19	25.00	Indeterminable
Rumex tunetanus	27.78	31.94	Indeterminable

Teucrium nablii	25.93	30.56	Indeterminable
Bituminaria tunetana	24.07	5.56	Indeterminable
Centaurea sicula subsp. *kroumirensis*	24.07	20.83	Indeterminable
Dianthus cintranus subsp. *byzacenus*	24.07	19.44	Indeterminable
Dianthus rupicola subsp. *hermaeensis*	24.07	26.39	Indeterminable
Elaeoselinum tunetanum	24.07	16.67	Indeterminable
Erinacea schoenenbergeri	24.07	11.11	Indeterminable
Galium afropusillum	24.07	27.78	Indeterminable
Galium olivetorum	24.07	23.61	Indeterminable
Helianthemum crassifolium subsp. *djeniense*	24.07	23.61	Indeterminable
Helianthemum virgatum subsp. *africanum*	24.07	20.83	Indeterminable
Lathyrus brachyodon	24.07	20.83	Indeterminable
Limonium boitardii	24.07	23.61	Indeterminable
Limonium byzacium	24.07	23.61	Indeterminable
Limonium cercinense	24.07	27.78	Indeterminable
Limonium clupeanum	24.07	27.78	Indeterminable
Limonium comosum	24.07	23.61	Indeterminable
Limonium confertum	24.07	25.00	Indeterminable
Limonium elfahsianum	24.07	27.78	Indeterminable
Limonium formosum	24.07	23.61	Indeterminable
Limonium hipponense	24.07	23.61	Indeterminable
Limonium intricatum	24.07	27.78	Indeterminable
Limonium kairouanum	24.07	27.78	Indeterminable
Limonium korbousense	24.07	27.78	Indeterminable
Limonium lacertosum	24.07	23.61	Indeterminable
Limonium menigense	24.07	27.78	Indeterminable
Limonium neapolense	24.07	27.78	Indeterminable
Limonium oblanceolatum	24.07	23.61	Indeterminable
Limonium punicum	24.07	23.61	Indeterminable
Limonium pyramidatum	24.07	27.78	Indeterminable
Limonium rubescens	24.07	23.61	Indeterminable
Limonium serratum	24.07	27.78	Indeterminable
Limonium tacapense	24.07	23.61	Indeterminable
Limonium thaenicum	24.07	27.78	Indeterminable
Limonium tritonianum	24.07	23.61	Indeterminable
Limonium xerophylum	24.07	27.78	Indeterminable
Limonium zembrae	24.07	27.78	Indeterminable
Limonium zeugitanum	24.07	27.78	Indeterminable
Odontites citrinus	24.07	9.72	Indeterminable

Sideritis tunetana	24.07	22.22	Indeterminable
Silene barrattei	24.07	20.83	Indeterminable
Thymelaea sempervirens	24.07	22.22	Indeterminable
Trifolium tunetanum	24.07	20.83	Indeterminable
Clematis tunisiatica	22.22	20.83	Indeterminable
Delphinium sylvaticum subsp. *ichkeulianum*	22.22	27.78	Indeterminable
Astragalus asterias subsp. *aristidis*	20.37	19.44	Indeterminable
Crepis juvenalis	20.37	5.56	Indeterminable
Lomelosia robertii	20.37	27.78	Indeterminable
Sixalix lehoueroui	20.37	27.78	Indeterminable
Sixalix thysdrusiana	20.37	18.06	Indeterminable
Teucrium radicans	20.37	27.78	Indeterminable
Linaria multicaulis subsp. *multicaulis*	16.67	13.89	Indeterminable
Ophrys omegaifera subsp. *hayekii*	16.67	25.00	Indeterminable
Ophrys pseudomigoutiana	16.67	13.89	Indeterminable
Ophrys x carpitana	16.67	27.78	Indeterminable
Serapias × debelairii	16.67	23.61	Indeterminable
Anarrhinum brevifolium	14.81	11.11	Indeterminable
Bellevalia dolichophylla	14.81	27.78	Indeterminable
Bellevalia galitensis	14.81	27.78	Indeterminable
Erysimum teppneri	14.81	22.22	Indeterminable
Hyacinthoides kroumiriensis	14.81	15.28	Indeterminable
Linaria aegyptiaca subsp. *glutinosa*	12.96	27.78	Indeterminable
Linaria cossonii	12.96	20.83	Indeterminable
Linaria paradoxa	12.96	25.00	Indeterminable
Linaria pseudoviscosa	12.96	27.78	Indeterminable
Linaria reflexa subsp. *doumetii*	12.96	27.78	Indeterminable
Linaria virgata subsp. *syrtica*	12.96	22.22	Indeterminable
Linaria virgata subsp. *tunetana*	12.96	25.00	Indeterminable
Arabis tunetana	7.41	12.50	Indeterminable
Festuca scholziana	7.41	18.06	Indeterminable
Oncostema maireana	7.41	18.06	Indeterminable
Poa lehoueroui	7.41	16.67	Indeterminable
Sporobolus tourneuxii	7.41	27.78	Indeterminable
Stipa alba	7.41	27.78	Indeterminable

References

Krigas, N.; Tsoktouridis, G.; Anestis, I.; Khabbach, A.; Libiad, M.; Megdiche-Ksouri, W.; Ghrabi-Gammar, Z.; Lamchouri, F.; Tsiripidis, I.; Tsiafouli, M. Exploring the potential of neglected local endemic plants of three

Mediterranean regions in the ornamental sector: Value chain feasibility and readiness timescale for their sustainable exploitation. Sustainability. **2021**, *13*, 2539.
